# CRISPR technology in human diseases

**DOI:** 10.1002/mco2.672

**Published:** 2024-07-29

**Authors:** Qiang Feng, Qirong Li, Hengzong Zhou, Zhan Wang, Chao Lin, Ziping Jiang, Tianjia Liu, Dongxu Wang

**Affiliations:** ^1^ Laboratory Animal Center College of Animal Science Jilin University Changchun China; ^2^ Research and Development Centre Baicheng Medical College Baicheng China; ^3^ School of Grain Science and Technology Jilin Business and Technology College Changchun China; ^4^ Department of Hand and Foot Surgery The First Hospital of Jilin University Changchun China

**Keywords:** clinical research, CRISPR–Cas9, gene editing technology, gene therapy, human diseases, sickle cell disease

## Abstract

Gene editing is a growing gene engineering technique that allows accurate editing of a broad spectrum of gene‐regulated diseases to achieve curative treatment and also has the potential to be used as an adjunct to the conventional treatment of diseases. Gene editing technology, mainly based on clustered regularly interspaced palindromic repeats (CRISPR)–CRISPR‐associated protein systems, which is capable of generating genetic modifications in somatic cells, provides a promising new strategy for gene therapy for a wide range of human diseases. Currently, gene editing technology shows great application prospects in a variety of human diseases, not only in therapeutic potential but also in the construction of animal models of human diseases. This paper describes the application of gene editing technology in hematological diseases, solid tumors, immune disorders, ophthalmological diseases, and metabolic diseases; focuses on the therapeutic strategies of gene editing technology in sickle cell disease; provides an overview of the role of gene editing technology in the construction of animal models of human diseases; and discusses the limitations of gene editing technology in the treatment of diseases, which is intended to provide an important reference for the applications of gene editing technology in the human disease.

## Graphical abstract

Gene editing is an evolving genetic engineering technology that shows great promise for application in a wide range of human diseases, not only for its therapeutic potential, but also for the construction of animal models of human diseases. This review paper describes the application of gene editing technology in hematological disorders, solid tumors, immune disorders, ophthalmic disorders, and metabolic disorders; highlights the therapeutic strategy of gene editing technology in sickle cell disease; outlines the role of gene editing technology in constructing animal models of human diseases; and discusses the limitations of gene editing technology in the treatment of diseases.

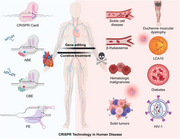

## 1 INTRODUCTION

Gene editing is an important genetic engineering technique to modify the target genes of organisms. Gene editing technology can directly perform site‐directed knockout, insertion, and mutation of specific target sequences in genetic material, changing the DNA sequence to induce specific gene inactivation or repair damaged genes to achieve precision medicine.^1^ Currently, gene editing technology has evolved from zinc finger nucleases (ZFNs) and transcription activator‐like effector nucleases (TALENs) to the clustered regularly interspaced palindromic repeats (CRISPR)–CRISPR‐associated protein (Cas) system.^2^ ZFNs and TALENs are limited by complex vector construction, low transfection efficiency, and multiple mutations, promoting the in‐depth development and research of the CRISPR–Cas system.^3^

The CRISPR–Cas system is a prokaryotic adaptive immunity mechanism that uses RNA‐directed endonucleases to cut foreign DNA or genetic material. The CRISPR–Cas system has emerged as the leading genome editing technology due to its simplicity, efficiency, and targeted ability to be precisely programmed or controlled.^4^ It has tremendous potential for applications in biotechnology and therapeutic interventions for diseases. CRISPR–Cas9, CRISPR–Cas12, and CRISPR–Cas13 are the main research tools of the CRISPR gene editing system.^5^ Base editors and prime editors were also used for experimental studies. The CRISPR–Cas system is extensively used in gene editing, epigenetic engineering, transcriptome engineering, gene regulation, nucleic acid detection, and RNA imaging.^6^

Genome editing technology has revolutionized gene therapy by shifting the focus from delivering exogenous transgenes to directly modifying the human genome sequence. Gene editing technology has driven the clinical use of gene and cell therapies in human disease, considering its great potential to correct genetic diseases and enhance cellular therapies.^7^ In the human disease field, gene editing technology can effectively treat some genetically induced or congenitally inherited hematologic diseases, solid tumors, immune diseases, eye diseases, and metabolic diseases.^8^, ^9^ However, gene therapy faces challenges due to the generation of off‐target effects during the gene editing process and the safety of the delivery vector. Animal models are important tools for studying human diseases and verifying genome editing efficiency in vivo is crucial for assessing its effectiveness.^10^ Animal models of human diseases offer a more intuitive assessment of the effectiveness and safety of gene therapy approaches, which is conducive to the development and clinical transformation of gene therapy strategies.

This review focuses on the progress of the CRISPR system; explores the advances, challenges, and potential therapeutic implications of some of the human diseases that have received United States Food and Drug Administration (US FDA) approval for treatment based on gene editing systems and emphasizes the significance of using animal models in preclinical studies of human diseases. It focuses on an in‐depth discussion of gene therapy for sickle cell disease (SCD) and also briefly discusses the limitations of gene editing related to off‐target effects and delivery in disease treatment.

## 2 OVERVIEW OF THE CRISPR–Cas SYSTEM

CRISPR and Cas proteins, comprising the adaptive immune system of prokaryotes,^11^, ^12^ represent the most versatile tool for genome editing in molecular biology.^13^, ^14^ The CRISPR–Cas system uses nonhomologous end‐joining (NHEJ) and homology‐dependent repair (HDR) for DNA editing.^7^ The NHEJ pathway fixes double‐stranded DNA breaks (DSBs) via nonspecific insertions or deletions that cause shift mutations or knockouts at the DSB site. In contrast, the HDR pathway introduces the desired sequence using an exogenous DNA repair template for genomic recombination.^15^ The CRISPR–Cas system depends on a CRISPR RNA (crRNA) for guidance and specific targeting or a guide RNA (gRNA).^7^

**FIGURE 1:**
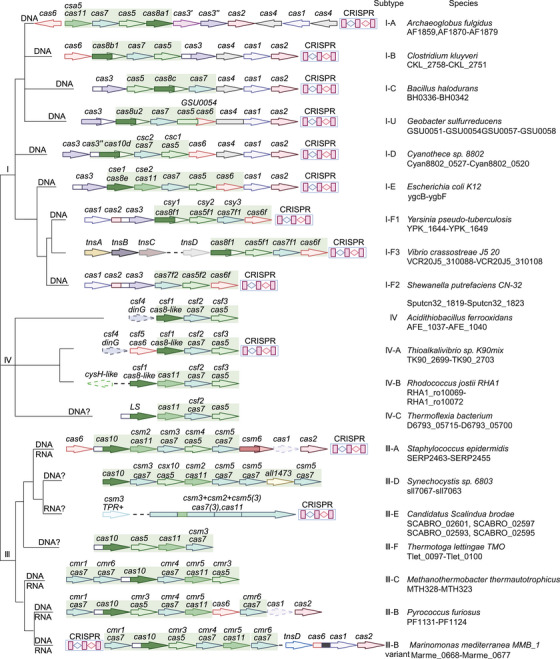
Representative/typical CRISPR–Cas loci and selected unique variants for class 1 CRISPR–Cas system isoforms. The effector module is indicated by light green shading. The dendrogram on the left shows the evolutionary relationships possible between the different types and subtypes of CRISPR–Cas systems, and the right column indicates the organism and the corresponding gene range. Image created in BioRender.com.

**FIGURE 2:**
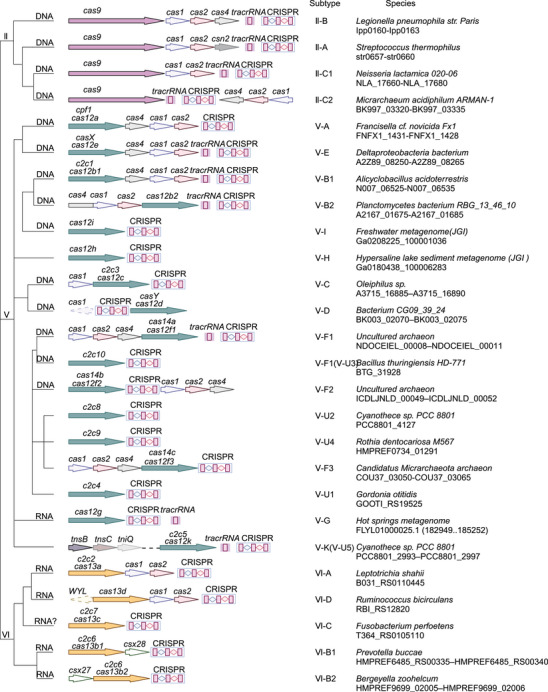
Representative/typical CRISPR–Cas loci for class 2 CRISPR–Cas system isoforms. The left dendrogram shows possible evolutionary relationships between different types and subtypes of CRISPR–Cas systems, while the right column indicates organisms and corresponding gene ranges. Image created in BioRender.com.

Over the last few years, several tools for gene editing for both simple and complex genomes have been discovered. The CRISPR–Cas system has complex and practical features as a gene editing tool.^16^ According to its effector proteins, CRISPR–Cas systems are classified into two classes that are further subdivided into six types (I–VI) and 33 subtypes.^17^ Class 1 systems (types I, III, and IV) usually encode numerous Cas effectors (Figure 1), while class 2 systems (types II, V, and VI) encode only one Cas effector protein (Figure 2). The class 2 CRISPR–Cas system for genome editing has established itself as an attractive option during the development of optimized genome editing technologies due to the simple structural design of its effector complexes (Table 1). The CRISPR–Cas9 system stands out as the best tool available for gene editing. Novel CRISPR–Cas systems, like SpCas9‐NG, base editing, xCas9, Cpf1, Cas13, and Cas14, have been gradually applied in gene editing research.^5^ Base and prime editing (PE) are currently advanced genome editing methods that directly modify target bases during genome and transcriptome editing.^15^

**TABLE 1:** Overview of Class II CRISPR/Cas systems and their main direct homologs or engineered variants.

Type	CRISPR–Cas system	Origin	Size (aa)	PAM/PFS	Target type	Application	References
II	SpCas9	*Streptococcus pyogenes*	1368	NGG	DNA	The first direct homolog of Cas9 capable of targeted mutagenesis in human cells. Guide RNA can be programmed as individual transcripts to target and cleave any dsDNA sequence of interest.	18
	eSpCas9	*Streptococcus pyogenes*	1368	NGG	DNA	Reduction in genome‐wide off‐target cutting	19
	VRER SpCas9	*Streptococcus pyogenes*	1368	NGCG	DNA	Increased opportunity for efficient HDR using the CRISPR–Cas9 platform, generating NHEJ‐mediated indels in small genetic elements	20
	VQR SpCas9	*Streptococcus pyogenes*	1368	NGAN/NGNG	DNA	Similar (or better) genome‐wide specificity compared with spCas9	20
	EQR SpCas9	*Streptococcus pyogenes*	1368	NGAG	DNA	Expanded targeting range of SpCas9	20
	SpCas9n(D10A)	*Streptococcus pyogenes*	1368	NGG	DNA	Reduction of off‐target mutations	21
	SpCas9‐HF1	*Streptococcus pyogenes*	1368	NGG	DNA	A high‐fidelity variant that reduces off‐target editing and improves specificity	22
	HypaCas9	*Streptococcus pyogenes*	1368	NGG	DNA	Exhibits high genome‐wide specificity without affecting human cell targeting activity	23
	HiFi Cas9	*Streptococcus pyogenes*	1368	NGG	DNA	Retains high on‐target activity while reducing off‐target editing	24
	evoCas9	*Streptococcus pyogenes*	1368	NGG	DNA	Provides very high specificity and produces fewer off‐target sites compared with wild‐type nucleases	25
	xCas9	*Streptococcus pyogenes*	1368	NG, GAA, GAT	DNA	Expanded PAM compatibility, high DNA specificity, and low genome‐wide off‐target activity	26
	SpCas9‐NG	*Streptococcus pyogenes*	1368	NG	DNA	Induced insertional deletion at endogenous target sites carrying NG PAM in human cells	27
	Evolved SpCas9 variants	*Streptococcus pyogenes*	1368	NRNH	DNA	SpCas9–NRRH, SpCas9–NRTH, and SpCas9–NRCH expand the targeting range of gene editing, enabling base editing of previously inaccessible pathogenic SNPs	28
	Sniper‐Cas9	*Streptococcus pyogenes*	1368		DNA	Having extended or truncated sgRNAs further reduces off‐target activity and allows DNA‐free genome editing	29
	Split‐Cas9	*Streptococcus pyogenes(residues 56–714)*	–	NGG	DNA	Maintain the cutting activity of natural enzymes	30
	SpG	*Streptococcus pyogenes*	1368	NGN	DNA	Expanding the scope of gene editing	31
	SpRY	*Streptococcus pyogenes*	1368	NRN/NYN	DNA	Can identify almost all PAM sequences in the genome and can eliminate these off‐target effects without affecting gene editing activity	31
	SaCas9	*Staphylococcus aureus*	1053	NNGRRT	DNA	Potential for efficient, specific and well tolerated in vivo genome editing applications	32
	KKH SaCas9	*Staphylococcus aureus*	1053	NNNRRT	DNA	This variant increases the target range of SaCas9 by nearly two to four times in random DNA sequences.	33
	SaCas9‐HF	*Staphylococcus aureus*	1053	NNGRRT	DNA	Displayed higher genome‐wide specificity than wild‐type SaCas9	34
	efSaCas9	*Staphylococcus aureus*	1053	NNGRRT	DNA	Has higher fidelity, does not affect cutting activity, and reduces off‐target cutting	35
	ScCas9	*Streptococcus canis*	1375	NGG	DNA	Both as an alternative genome editing tool and as a functional platform to discover new Streptococcus PAM specificities	36
	FnCas9	*Francisella novicida*	1629	NGG	DNA	FnCas9 is one of the largest direct homologs of Cas9, complexed with guide RNA and its PAM‐containing DNA targets.	37
	Nm(e)Cas9	*Neisseria meningitidis*	1082	NNNNGATT	DNA	The use of sgRNA to guide gene editing activities increases the sequence environment available for RNA‐guided genome editing due to its unique protospacer‐adjacent pattern.	38
	Nme2Cas9	*Neisseria meningitidis*	1082	NNNNCC	DNA	Nme2Cas9 combines the compatibility of an all‐in‐one AAV with superior intracellular editing precision to edit a high density of genomic loci.	39
	St(h)1Cas9	*Streptococcus thermophilus*	1121	NNAGAAW	DNA	Enables efficient and precise DNA break and insertion deletion formation without any off‐target effects	40
	St(h)3Cas9	*Streptococcus thermophilus*	1409	NGGNG	DNA	Achieved effective genomic targeting in human cells	41
	GeoCas9	*Geobacillus stearothermophilus*	1087	NNNNCRAA	DNA	Catalyzing RNA‐guided DNA cleavage at high temperatures extends the temperature range for CRISPR–Cas9 applications	42
	CjCas9	*Campylobacter jejuni*	984	NNNNACAC	DNA	CjCas9 is much smaller than SpCas9 or SaCas9, with better editing efficiency and specificity.	43
	BlatCas9	*Brevibacillus laterosporus*	1092	NNNNCNAA	DNA	A compact Cas9 nuclease with specificity to be improved due to its ability to tolerate dinucleotide mismatches at positions 1–11.	44
	SauriCas9	*Staphylococcus auricularis*	1061	NNGG	DNA	Gene size is small and can be packaged into AAV along with gRNA with simple PAM sequences.	45
	SauriCas9‐KKH	*Staphylococcus auricularis*	1061	NNAG	DNA	Expanded targeting range	45
	SmacCas9	*Streptococcus macacae*	1366	NAAN	DNA	Minimal adenine dinucleotide PAM specificity is maintained for efficient and accurate gene editing activity.	46
	TdCas9	*Treponema denticola*	1423	NAAAAN	DNA	A larger Cas9 protein that may differ from smaller family members	47
	HiFi‐Sc++	*Streptococcus canis*		NNG		Possesses robust DNA cleavage activity and minimal off‐target activity	48
V	FnCas12a(FnCpf1)	*Francisella novicida U112*	1300	TTN	DNA	Precise deletion of single or double genes is possible, and when no template for HDR is available, random‐sized DNA deletion is achieved through the reconstituted NHEJ pathway via FnCpf1‐induced DSB repair.	49
	AsCas12a(AsCpf1)	*Acidaminococcus sp. BV3L6*	1307	TTTN	DNA	The RuvC nuclease structural domains of Cas9 and Cpf1 are homologous.	50
	enAsCas12a	*Acidaminococcus sp*.	1307	TTYN/VTTV/TRTV	DNA	Expanded targeting range and greatly improved cleavage efficiency at lower temperatures.	51
	enAsCas12a‐HF1	*Acidaminococcus sp*.	1307	TTTN	DNA	Higher gene editing activity	51
	AsCas12a Ultra	*Acidaminococcus sp*.	1307	TTTN	DNA	Higher and more powerful editing efficiency	52
	AsCas12a‐RVR	*Acidaminococcus sp*.	1307	TATV	DNA	High DNA targeting specificity and expanded targeting of Cpf1	53
	AsCas12a‐RR	*Acidaminococcus sp*.	1307	TYCV	DNA	High DNA targeting specificity and expanded targeting of Cpf1	53
	LbCas12a(LbCpf1)	*Lachnospiraceae bacterium ND2006*	1228	TTN	DNA	While Cas9 produces a cleavage product that is blunt‐ended, Cpf1 staggers the cleavage, leaving a 5’‐nucleotide overhang at the distal end of the PAM site. Cpf1 uses a T‐rich PAM sequence, which differs from Cas9's preference for G‐rich PAMs.	54
	LbCas12a‐RVRR	*Lachnospiraceae bacterium*	1228	TNTN/TACV/TTCV/CTCV/CCCV	DNA	Expanded PAM, retaining a slightly higher preference for ‐3'T' nucleotides	55
	impLbCas12a	*Lachnospiraceae bacterium*	1228	TTTV/TCCV/CCCV/TATCC/TACV	DNA	Extended target space and high activity at lower temperatures	55
	Lb2 Cas12a(Lb2 Cpf1)	*Lachnospiraceae bacterium MA2020*	1206	TTNN	DNA	Smaller than LbCas12a, with higher editing efficiency and off‐target activity	56
	Lb2‐KY	*Lachnospiraceae bacterium MA2020*	1206	NTTN	DNA	High editing efficiency and extensive PAM	57
	PiCas12a	*Prevotella ihumii*	–	TXTL	DNA	Identify PAMs with multiple G's in their PAM	58
	PdCas12a	*Prevotella disiens*	–	ATTTC	DNA	Easier to drive transcriptional silencing compared with DNA cleavage	58
	HkCas12a	*Helcococcus kunzii ATCC 51366*	–	TXTL and C‐rich PAM	DNA	Ability to efficiently identify C‐rich motifs unrelated to other well‐characterized Cas12a nucleases	58
	AaCas12b	*Alicyclobacillus acidiphilus*	1129	TTN	DNA	Relatively small size, higher stability in human plasma, high specificity	59
	AkCas12b	*Alicyclobacillus kakegawensis*	1147	TTTN	DNA	Can cleave target DNA	59
	BhCas12b v4	*Bacillus hisashii*	1108	ATTN	DNA	High specificity and high efficiency of DNA cleavage	60
	BthCas12b	*Bacillus thermoamylovorans*	–	ATTN	DNA	Low editing efficiency	61
	AacCas12b	*Alicyclobacillus acidoterrestris*	1277	TTN	DNA	Cleaves target DNA between 37 and 60°C and shows temperature‐dependent cleavage activity	62
	Cas12c1	*–*	1302	TG	DNA	An efficient and high‐fidelity DNA targeting tool	63
	Cas12c2	*–*	1218	TN	DNA	With the smallest PAM, it offers greater flexibility and a wide range of targets.	64
	OspCas12c	*Oleiphilus sp. HI0009*		TG	DNA	Has RNA‐guided dsDNA interference activity	64
	Cas12d (CasY)	*Uncultivated archaea*	1126	TA	DNA	Possesses DNA interference activity	65
	DpbCas12e(DpbCasX)	*Deltaproteobacteria*	986	TTCN	DNA	A hybrid of Cas9, Cas12a, and novel RNA folding and protein structures, it belongs to a third distinct class of CRISPR systems capable of targeted genome regulation and editing.	66
	PlmCas12e (PlmCasX)	*Planctomytes*	984	TTCN	DNA	PlmCasX has the same or higher genome editing activity in mammalian cells compared with pbCasX.	67
	Un1Cas12f1 (Cas14a1)	*–*	400–700	No PAM constraints	DNA	Specialized targeting and cleavage of ssDNA in a PAM nondependent manner	68, 69
	CasMINI	*–*	529	TTTR	DNA	Robust gene editing possible in mammalian cells	70
	AsCas12f1	*Acidibacillus sulfuroxidans*	422	NTTR	DNA	Preferential dsDNA target binding and cleavage at higher temperatures (45–55°C)	71
	SpCas12f1	*Syntrophomonas palmitatica*	497	TTC	DNA	Mediated genome editing in eukaryotic cells	71
	Cas12g	*–*	720‐830	No PAM constraints	DNA	With side branch RNase and single chain DNase activity	72
	Cas12h	*–*	870‐933	RTR	DNA	Large truncation of the N‐terminal region responsible for PAM recognition and DNA unfolding	72
	Cas12i	*–*	1033‐1093	TTN	DNA	Exhibited significantly different CRISPR RNA spacer complementary and noncomplementary strand cleavage efficiencies	72
	Cas12j(CasΦ)	*–*	757	TBN	DNA	Molecular weight is only half that of Cas9 and Cas12a genome editing enzymes, providing advantages for cellular delivery.	73
	Cas14	*Uncultivated archaea*	400‐700	No PAM constraints	ssDNA	The smallest RNA‐directed nuclease ever discovered and the only identified PAM‐independent DNA‐targeting Cas.	74
VI	LwaCas13a	*Leptotrichia wadei*	1152	No significant PFS motif	RNA	Allows programmable tracking of transcripts in live cells	75
	LshCas13a	*Leptotrichia shahii*	1389	3′ H PFS motif	RNA	Cleaves RNA through conserved basic residues within two HEPN structural domains, in contrast to the catalytic mechanism of other known RNA enzymes of the CRISPR–Cas system	76
	Cas13bt	*–*	775–804	5′ D (A/G/T) PFS	RNA	Can be used to generate compact REPAIR and RESCUE constructs compatible with AAV delivery	77
	PspCas13b	*Prevotella sp. P13‐5*	1092	No PFS constraints	RNA	Absence of PFS constraints that interfere with mammalian cells	78
	PbCas13b	*Prevotella buccae*	1127	NAN/NNA 3′ PFS	RNA	In vitro cleavage of targeted single‐stranded RNA	79
	BzCas13b	*B. zoohelcum*	∼1150	NAN/NNA 3′ PFS	RNA	The single‐protein effector cleaves single‐stranded RNA through the HEPN structural domain and exhibits side‐branch RNase activity.	79
	FpeCas13c	*Fusobacterium perfoetens*	1121	Unknown	RNA	The least characterized Cas13 enzyme may use a different mechanism than other Cas13 enzymes.	80
	RfxCas13d(CasRx)	*Ruminococcus flavefaciens*	967	No PFS constraints	RNA	One of the most compact single effect Cas enzymes with high efficiency and specificity	81
	Es Cas13d	*Eubacterium siraeum*	954	No PFS constraints	RNA	Small size and minimal targeting restrictions extend the RNA editing tools	82
	Rsp Cas13d	*Ruminococcus sp*.	∼930	No PFS constraints	RNA	With lateral branch RNase activity	82
	Cas13X	*–*	1775	No PFS constraints	RNA	A compact RNA interference tool with relatively high efficiency and specificity	83
	Cas13Y	*–*	1790	–	–	Small length	83

N represents A/C/G/T; D represents A/G/T; R represents A/G; Y represents C/T; W represents A/T; H represents A/C/T; B represents C/G/T; V represents A/C/G.

Abbreviations: aa, amino acids; PAM, protospacer adjacent motif; PFS, protospacer flanking sequence.

### 2.1 CRISPR–Cas9

**FIGURE 3:**
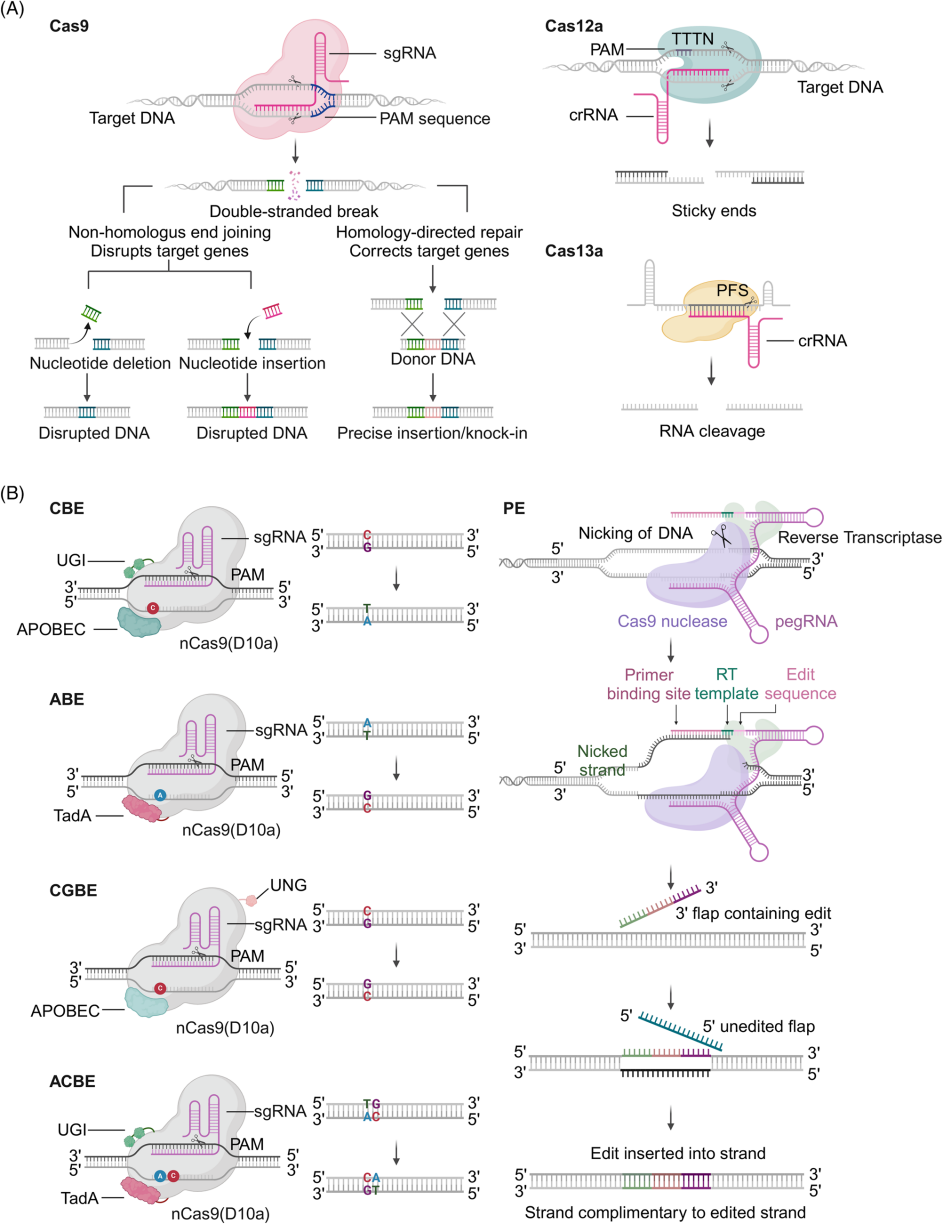
Schematic diagram of representative Cas proteins and BE tools of different families of class 2 CRISPR–Cas systems used for genome engineering. (A) Schematic diagram of the structure and mechanism of SpCas9, Cas12a, and Cas13a. (B) Schematic diagrams of the structures and mechanisms of CBE, ABE, CGBE, A&C‐BEmax, and PE. sgRNA, single guide RNA. N represents A/C/G/T. Image created in BioRender.com.

CRISPR–Cas9 is an important gene editing tool for gene knockout, knock‐in, repression and activation, multiple editing, and genomic function screening.^84^, ^85^ *S. pyogenes* Cas9 (SpCas9; 1368 amino acids) was the first *Streptococcus* protein used outside prokaryotic cells for mammalian cell genome editing (Figure 3A).^86^ The key limitation of genome editing using the CRISPR–Cas9 system is the need for a protospacer adjacent motif (PAM) pattern at the target site. SpCas9 DNA targeting depends on PAM 5’‐NGG (N represents any nucleotide).^87^ Studies have established the SpCas9 high‐fidelity variant SpCas9‐HF1, which has comparable targeting to wild‐type SpCas9 without detectable genome‐wide off‐target effects, making it a suitable nuclease for research and treatment applications.^22^ Developing an engineered Cas9 variant with a lower PAM restriction than NGG is one strategy to solve the PAM restriction. Variant xCas9 has expanded PAM compatibility to recognize PAM sequences that include NG, GAA, and GAT.^26^ The activity of off‐targeting is minimal during the targeting of non‐NGG‐PAM genomic loci.^26^

The development of engineered Cas9 variants has expanded the range of DNA targeting by CRISPR systems.^26^ Identifying Cas9 homologs with different PAM sequences provides greater target site selection for gene editing applications. *Staphylococcus aureus* Cas9 (SaCas9; 1053 amino acids) recognizes a PAM of 5'‐NNGRRT (R represents A or G) and has similar gene editing efficiency to SpCas9 with a smaller size.^32^ Currently, the smallest Cas9 is CasX (980 amino acids).^65^ Cas9‐NG recognizes the smallest PAM (NG), expanding the available targets for genome editing in human cells and showing significant potential for genome editing, base editing, and genome regulation.^88^ Another effect of the Cas9 variant design is to increase gene editing targeting specificity. SpCas9 (eSpCas9) variants with enhanced specificity minimize off‐target effects; maintain robust, targeted cleavage; and are widely used for targeted genome editing applications that require high specificity.^19^

### 2.2 Base editing

Base editing (BE) is one of the latest advances in genome editing. Using BEs to introduce temporary RNA or permanent DNA base changes shows great promise in curing many genetic diseases.^15^ The largest class of human disease‐causing mutations are point mutations, known as single nucleotide polymorphisms (SNPs).^89^ Since DSBs generated by nucleases such as Cas9 result in unwanted by‐products of point mutations such as insertions and deletions, translocations, and rearrangements, BE technology arose at a historic moment.^89^ Base editing is a gene editing technique targeting single nucleotides.^90^ Base editing systems could specifically edit the bases on the target DNA,^91^, ^92^ eliminating the requirement for either donor DNA templates, DSBs, HDR, or NHEJ,^93^, ^94^ minimizing the formation of DSB‐related by‐products.^95^ A variety of BE systems have been developed, and several of the more common BE editors are briefly described below (Table 2).

**TABLE 2:** A brief summary of base editors.

Base editor	PAM	Base conversion	References
BE1	NGG	C‐to‐T/G‐to‐A	94
BE2	NGG	C‐to‐T/G‐to‐A	94
BE3	NGG	C‐to‐T/G‐to‐A	94
SaBE3	NNGRRT	C‐to‐T/G‐to‐A	95
BE4	NGG	C‐to‐T/G‐to‐A	95
SaBE4	NNGRRT	C‐to‐T/G‐to‐A	95
dCpf1‐BE (Cas12a‐BE)	TTTV	C‐to‐T	96
eA3A‐BE3	NGG	C‐to‐T	97
CGBE	NGG	C‐to‐G/C‐to‐A	98
GBE	NGG	C‐to‐G/C‐to‐A	99
ABE	NGG	A‐to‐G/T‐to‐C	93
AYBEv3	NGG	A‐to‐C/A‐to‐T	100
IBE	NGG	A‐to‐G	101
ABE8e	NGG	C‐to‐T/G‐to‐A/A‐to‐G/T‐to‐C	102, 103
ABEmax	NGG	A‐to‐G/T‐to‐C	104
ABE7.10	NGG	A‐to‐G/T‐to‐C	105
ABE9	NGG	A‐to‐G	106
Target‐ACEmax	NGG	C‐to‐T/A‐to‐G	107
A&C‐BEmax	NGG	C‐to‐T/A‐to‐G	108
AGBE	NGG	C‐to‐G/C‐to‐T/C‐to‐A/A‐to‐G	109

N represents A/C/G/T; R represents A/G; V represents A/C/G.

Abbreviation: PAM, protospacer adjacent motif.

#### 2.2.1 Cytosine BEs

The cytosine BE (CBE) system converts codons CAA, CAG, CGA, and TGG into a stop codon by mediating the conversion of the target site C to T.^110^ BE3, BE4, and dCpf1‐BE (d means catalytically dead) use Cas9 or Cpf1 variants to recruit cytidine deaminases to produce specific C to T or G to A changes via DNA mismatch repair (MMR) pathways (Figure 3B).^95^, ^96^ The BE3 system links rat cytidine deaminase apolipoprotein B mRNA editing enzyme catalytic subunit 1 (APOBEC1) with the Cas9 cleavage enzyme and uracil glycosylase inhibitor.^111^ BE3 editing is confined to a five‐nucleotide (nt) editing window. It is effective in correcting various point mutations associated with human disease but is less efficient in the GC environment.^112^ Fourth‐generation BEs BE4 (BE derived from SpCas9) and SaBE4 (BE4 derived from SaCas9) offer greater efficiency with higher product purity than BE3.^95^ BE3‐Gam, SaBE3‐Gam, BE4‐Gam, and SaBE4‐Gam improve product purity by minimizing the formation of unneeded insertions and deletions in the base editing process.^95^ However, the target range of BE is restricted by the sequence of the G/C‐rich PAM it contains. The CRISPR–Cpf1‐based BE recognizes PAM sequences rich in T and catalyzes the C‐T transition with low insertion‐deletion and off‐target editing levels by combining APOBEC1 with the Cpf1 of *Aureobasidium pullulans*.^96^ The base targeting horizon expands after fusing APOBEC1 with the catalytically inactive dLbCas12a, which has a six base pair (bp) activity window and can recognize T‐rich PAMs.^96^ The xCas9 variant facilitates various non‐NGG PAMs by BE or DNA cleavage, requiring highly accurate matching between gRNA and target sequences.^97^ CBE was constructed by replacing Cas9 with xCas9 in the BE3 vector, which can edit target PAM sequences of NGN, GAT, and GAA.^26^, ^110^, ^113^ The BE comprising the Cas9 incision enzyme and human apolipoprotein B mRNA editing enzyme catalytic subunit 3A (APOBEC3A; A3A‐PBE) has a 17 nt editing window and can effectively achieve C‐T conversion.^114^ Engineered A3A (eA3A) domains can reduce bystander mutations and off‐target effects. Indeed, eA3A‐BE3 can correct point mutations in some human diseases more accurately than BE3.^97^ Recently, a study has developed highly active minibase editors miABE and miCBE by fusing adenosine deaminase and cytidine deaminase structural domains, opening a new chapter for minibases in the field of DNA single‐base editing.^115^

#### 2.2.2 Glycosylase BEs

Based on CBEs, C‐to‐G BEs (CGBEs) or glycosylase BEs (GBEs) can realize the transversion between C to G and C to A (Figure 3B).^116^ CGBE1 induces C to G editing in human cells rich in AT sequences. CGBEs can be engineered for efficient and accurate base editing.^116^ GBEs comprise cytidine deaminase, the Cas9 incision enzyme, and uracil‐DNA glycosylase (UNG). The GBE APOBEC‐nickase‐Cas9 (nCas9)‐UNG can cause C‐to‐A conversion in *Escherichia coli* and C‐to‐G conversion in mammalian cells, enabling the targeting of G‐C pathogenic variants.^99^ The development of CGBEs and GBEs has extended the research of DNA damage repair mechanisms and BE applications.^99^, ^117^ BEs with C:G to G:C activity, comprising nCas9 fused with cytidine deaminase and a base excision repair protein, have been developed to target cytidine in the context of WCW (W represents A/T), ACC, or GCT sequences and the precise trinucleotide window of the target protosepta, with therapeutic potential to correct human genetic diseases.^98^ In other studies, the tRNA adenosine deaminase (TadA)‐8e was repurposed for cytosine conversion. An N46L variant was introduced into TadA‐8e to eliminate its adenine deaminase activity and construct the first new CGBE/CBE family of base codification Td‐CGBE/Td‐CBEs independent of the Activation‐induced deaminase/APOBEC (AID/APOBEC) deaminase family.^118^ Td‐CGBEs and Td‐CBEs enable efficient and accurate C‐G‐to‐G‐C editing with a very low insertion‐deletion effect and off‐target editing, which can potentially be used for the clinical transformation of gene therapy. More recently, a novel DNA BE, glycosylase‐based guanine BE, which enables efficient guanine single‐base editing and does not depend on deamidation reactions, has been successfully developed to further enrich the base editing toolkit.^119^

#### 2.2.3 Adenine BEs

About half of the known pathogenic SNPs are represented by C‐G to T‐A mutations. Correcting most disease‐associated human SNPs by converting A‐T bps to G‐C bps using base‐editing techniques is possible.^93^ Adenine BEs (ABEs) were developed by fusing TadA with the SpCas9 cleavage enzyme to induce A‐T to G‐C conversion, significantly expanding the scope of base editing (Figure 3B).^93^ About a quarter of the SNPs causing genetic diseases in humans require translocation of adenine bases such as A‐to‐T and A‐to‐C or the complementary chains of T‐to‐A and T‐to‐G. The development of adenine transversion BE AYBEv3 filled the gap that current BEs cannot efficiently perform A‐to‐C or A‐to‐T editing.^100^

Studies have developed deaminase‐embedded inlaid BEs (IBEs) based on ABE using protein engineering.^101^ IBEs induce the conversion of bases from A‐to‐G, positioning the deaminase domain in the interior of Cas9 to avoid the PAM restrictions.^101^ ABE8e was developed to pair with various Cas9 or Cas12 homologs to efficiently install natural mutations and greatly improve editing efficiency, improving the efficiency and adaptability of adenine editing.^102^, ^103^ ABEmax and mini‐ABEmax (TadA* fused with Cas9n) variants have been constructed to develop engineered ABEs with minimal RNA off‐target activity, retaining DNA‐targeted editing activity while reducing RNA editing activity.^104^

ABE7.10 has low efficiency in editing challenging sites in primary human cells. Several studies have developed ABE8s through the evolution of ABE7.10, which effectively recapitulate the natural alleles on the hemoglobin (Hb) subunit gamma 1 (HBG1) and 2 (HBG2) promoters of the γ‐Hb genes of human CD34^+^ cells to induce HbF persistence.^105^ Another study designed TadA7.10, a commonly used adenosine deaminase that has a D108Q mutation and can reduce the cytosine editing activity of ABEs, ABE8e, and ABE8s at target sites and is compatible with mutations that reduce off‐target RNA editing. This base‐specific editing tool for T‐C to T‐T or T‐C to T‐G conversions broadens the usefulness of BEs.^120^ ABEs pioneered the A‐G transition for base editing, but mutations and off‐target editing effects among bystanders may cause safety concerns for gene editing. ABE8e with an N108Q mutation has been found to reduce bystander editing of adenine and cytosine, so ABE9 with an editing window reduced to 1–2 nt almost eliminates the off‐target editing of cytosine and reduces DNA/RNA off‐target events to background level. Theoretically, it provides a targeted editing tool for correcting nearly 50% of genetic pathogenicity point mutations.^106^

#### 2.2.4 Dual ABEs and CBEs

Currently, available ABEs and CBEs can induce the modifications of only one type, limiting the scope of DNA alterations. Some studies have combined traditional CBE and ABE tools to develop two‐base gene editing tools. Simultaneous programmable adenine and cytosine editing allows both A‐to‐G and C to T substitutions to be introduced and minimizes RNA off‐target editing.^121^ Target‐ACEmax comprises a Cas9 notch enzyme, cytosine deaminase Petromyzon marinus cytidine deaminase 1 (PmCDA1), and adenosine deaminase TadA and has high C‐T and A‐G editing activity. Dual ABE and CBE (A&C‐BEmax) can achieve C‐T and A‐G conversion at the same target site and have high cytosine editing activity.^107^ Compared with the single‐BEs, A&C‐BEmax shows significantly improved base transition rate, product purity, and simultaneous A/C conversion activity (Figure 3B).^108^ Another study integrated a CGBE and ABE to develop the double deaminase‐mediated base editing system A‐to‐G BE (AGBE), which can introduce four types of base conversion in mammalian cells: C‐to‐G, C‐to‐T, C‐to‐A, and A‐to‐G.^109^ Compared with ABEs and CGBEs, AGBEs are efficient and safe, have a much broader editing window, have no apparent off‐target effects at the DNA or RNA level, and are useful for building mutant libraries containing more gene mutation types.^109^

### 2.3 Prime editing

Prime editing (PE) is a gene editing technique that enables arbitrary base substitutions, insertions, and deletions of DNA fragments.^122^ While BEs can effectively achieve four transition mutations and improve the efficiency of correcting point mutations, they cannot achieve eight transition mutations and accurate insertion or eliminate target gene fragments.^91^ Moreover, DNA BEs are not sufficiently specific for gene editing.^123^ Meanwhile, the PE system involves nCas9 (an inactivated HNH nuclease) ligated with reverse transcriptase (RT) and a modified PE gRNA (pegRNA).^122^ PEs have unique advantages over other BEs, mediating the targeted insertions, deletions, and transitions of all 12 potentially available base‐to‐base conversions in human cells without introducing either DSBs or donor DNA templates with high specificity (Figure 3B).^122^, ^124^, ^125^ PE makes it possible to correct mutations related to most types of human genetic diseases, greatly expanding the ability and application of genome editing and laying the basis of gene therapy for clinical applications.^122^

#### 2.3.1 PE1, PE2, PE3, and PE3b

The original PE1 is a wild‐type RT of Moloney murine leukemia virus (M‐MLV) ligated to the C‐terminus of the SpCas9 H840A cut enzyme via a flexible junction to extend the pegRNA binding site (PBS) to 8–15 bases, PE1 can mediate cellular single‐base transitions, insertions and deletions, but with low efficiency, with a maximum editing efficiency of only 0.7–5.5%.^122^ To improve the base editing efficiency of PE1, the study introduced five mutations, D200N, L603W, T306K, W313F, and T330p, into M‐MLV RT and constructed PE2. PE2 increased the editing efficiency to 1.6–5.1‐fold of that of PE1, and with a shortened sequence of the PBS, was able to perform more targeted insertions and deletions, and the study further showed that that the first base of pegRNA in the 3' extension is not a C also contributes to the editing efficiency of PE2. Since the newly synthesized DNA editing strand in PE2 needs to undergo an endogenous repair process before it can be copied to the complementary strand, the study added additional sgRNAs to the PE2 system for guiding the SpCas9 H840A incision enzyme to cleave the unedited strand, which further boosted the editing efficiency of PE3 to 1.5–4.2‐fold that of PE2, but since there will be two DNA strands at the same time nicks, PE3 has the probability of generating an indel. Studies optimized sgRNAs with spacers matching the edited strand to avoid editing the original allele, generating the PE3b BE, and PE3b significantly reduced the formation of insertion deletions whilst providing editing efficiencies similar to those of PE3. The study confirmed that PE3 was able to achieve HBB E6V mutations in HEK293T cells with 44% efficiency and less than 5% insertional deletions, suggesting that PE has the potential for use in the treatment of human diseases.

#### 2.3.2 PE4 and PE5

After the development of PE1, PE2, PE3, and PE3b, it was found that DNA MMR in the PE system is a major limiting factor for editing efficiency and increases the production of by‐products. The study optimized PE2 and PE3 using a transiently expressed dominant‐negative MMR protein (MLH1dn), which is less susceptible to MMR activity, and constructed PE4 (PE2+MLH1dn) and PE5 (PE3+MLH1dn), two editors that were 7.7‐fold and 2.0‐fold more efficient than the editing efficiencies of PE2 and PE3, respectively.^126^ The study further designed and optimized Cas9 proteins and nucleation signals, and so on, and synergistically applied PEmax and epegRNA with PE4 or PE5, which showed greater enhancement of editing efficiency for PE4 and PE5, and constructed PE4max and PE5max, which showed higher editing efficiencies in all seven mammalian cell types.

#### 2.3.3 Homology 3' extension‐mediated PE

Based on the developed PE editors, a homology 3' extension‐mediated PE (HOPE) has been developed. The two pegRNAs of HOPE target the positive and negative DNA strands, respectively, and perform precise gene editing at the same locus of the genome, which greatly improves the editing efficiency of PE, and achieves highly efficient editing in HEK293T and human colorectal cancer cells HCT116 editing. Compared with PE2 and PE3, the efficiency of HOPE base editing was higher than that of PE2, and the purity of the product was better than that of PE3, which extended the application prospect of PE editing. The study also provided an in‐depth investigation of factors such as the spacing of DNA double‐stranded PAMs and the length of PBS versus RT, which provided directions for the optimization of double pegRNAs.^127^

#### 2.3.4 Twin plasmid editor

Twin plasmid editor (twinPE) is a new version of PE technology that addresses the limitation of the initial PE to edit only a few dozen bps, enabling safe and precise insertion of complete genes into human cells.^128^ twinPE uses a plasmid editing protein and two pegRNAs to provide programmable DNA sequence replacement or excision at human endogenous genomic loci, enabling targeted DNA plasmid (>5000 bp) incorporation and reversal of 40 kb of the target sequence in human cells, expanding the ability to perform precise gene editing with few by‐products (12). In twinPE editing, each pegRNA directs the editing protein to create single‐stranded gaps in the DNA at different target sites in the genome, avoiding the generation of meaningless byproducts, and subsequently synthesizing the desired sequence between the gaps, thereby inserting, replacing, or deleting sequences of up to 800 bps. twinPE may act synergistically with another tool to make corrections or additions to large or complex human disease‐causing genes for correction or complementation.

#### 2.3.5 PE6

For further optimization of the PE system, the study used protein evolution and engineering to generate PE editors with smaller sizes and higher efficiency. The study first developed RTs different from M‐MLV RT and then used PE editing phage‐assisted sequential evolution to screen and evolve three RTs with smaller sizes, Gs RT, Ec48, and Tf1, to generate PE editors that are smaller than the previous PE editors. It was found that different RTs specializing in different types of PE editors helped to improve PE editing efficiency, and improved structural domains of Cas9 also had a positive effect on editing efficiency. The study obtained a series of variants that are superior to previous PE editors in terms of size (PE6a and PE6b), RT activity (PE6b, PE6c, and PE6d), and Cas9‐dependent editing efficiency (PE6e–PE6g) through the screening and modification of different RTs and the evolution of Cas9, and a series of variants that are more efficiently customized according to the actual needs of gene editing can be selected.^129^

#### 2.3.6 PE7

Considering that the PE system is currently a more recent gene editor, a study using a genome‐wide scale CRISPR interference screen (CRISPRi screening) identified the small RNA‐binding protein La (La protein) as a key regulator of PE efficiency. La binds polycytidylic acid at the 3′ end of RNA polymerase III transcriptions, and La was found to functionally interact with the 3′ end of polycytidylic acidic lead editing gRNA (pegRNAs). The study fused La proteins and LA(1–194) truncated proteins into PEmax and showed that fusion at the C‐terminal end of La(1–194) fusion construct (PEmax‐C) was the most efficient, and the study thus constructed the PE7 editor. The editing efficiency of PE7 in U2OS cells was significantly higher than that of PEmax, and the construction of PE7 provides new ideas for the optimization and development of PE editors.^130^

### 2.4 CRISPR–Cas12

Cas12 produces deletion products after editing the genome, significantly reducing the proportion of base insertions, and is suitable for clinical diseases where specific DNA fragments need to be deleted for therapeutic purposes (Figure 3A). Cas12a (Cpf1) is a type V class II single RNA‐guided nucleic acid endonuclease lacking trans‐activating crRNA that cleaves DNA by interleaved DSBs.^49^ Cas12a produces staggered ends compared with Cas9, which may increase the efficiency of NHEJ‐based gene insertion.^131^ Cas12a can cut crRNA arrays to produce its own crRNAs, helping to simplify concurrent genome editing with multiple crRNAs using a single custom crRNA array.^132^ The Cas12a from the genus *Acidaminococcus* sp. (AsCas12a) and *Lachnospiraceae bacterium* (LbCas12a) are the first direct Cas12a homologs shown to actively recognize the PAM sequence 5'‐TTTV upstream of the candidate sequence in mammalian cells.^49^ An enhanced AsCas12a variant (enAsCas12a) was designed to improve and enhance the genome editing activity of Cas12a.^51^ A high‐fidelity version of enAsCas12a (enAsCas12a‐HF1) was also designed to minimize off‐target effects.^51^ Using engineered crRNA with a complementary 20‐nt candidate sequence and a uridylate‐rich 3′‐highlight sequence facilitates Cas12a activity with high safety and specificity.^131^ In addition, Cas12a only requires a 42 nt crRNA.^133^ Cas12a is useful as a detection tool upon activation by double‐stranded DNA targeting. The unique function of Cas12a enriches the CRISPR toolbox.

### 2.5 CRISPR–Cas13

RNA has an essential and diverse role as a tool in biology. However, quantitative tools for manipulating and measuring RNA are more limited. A class 2 type VI CRISPR–Cas system with a Cas13‐related effector is promising as a tool for RNA targeting or binding.^6^, ^75^ The Cas13 protein from the bacterium *Leptotrichia shahii*, an RNA‐directed nuclease, is required to recognize the protospacer flanking sequence (PFS), use the RNA cleavage activity of the two nucleotide‐binding HEPN domains, and clear single‐stranded RNA targets carrying a complementary prototype spacer (Figure 3A).^76^ The Cas13a from *Leptotrichia wadei* (LwaCas13a) and Cas13b from the genus *Prevotella* P5‐125 (PspCas13b) do not require PFS identification.^75^, ^78^ The catalysis‐inactive Cas13a variant dCas13A retains the ability to bind to target RNA for live cell imaging of RNA, making it a flexible platform for studying mammalian cellular RNA and therapeutics.^76^ Like RNA‐targeting Cas9 (RCas9), dCas13a was used as a target for mRNA and visually formed stress particles.^76^

### 2.6 Other major members of the CRISPR–Cas system

Cas14 is a very compact series of RNA‐directed nucleases (400–700 amino acids) divided into three subgroups: Cas14a, Cas14b, and Cas14c.^69^ Cas14a is the smallest RNA‐mediated Cas protein and does not require the presence of a PAM near the target site. The Cas14‐DETECTR detection method has been developed based on its ssDNA cutting activity, which has the potential to diagnose human diseases.^134^ Type III‐A/B Cas10 proteins are typically activated after binding to homologous target RNA to exert DNase or cyclic oligo‐adenylate synthetase activity.^135^ The crRNA of CRISPR–Cas10 does not recognize the target DNA sequence directly. Instead, it matches the transcribed pre‐mRNA while the target DNA is transcribed.^136^ Unlike conventional class 1 type III systems, the III‐E CRISPR–Cas system has four Cas7‐like structural domains and a Cas11‐like structural domain III‐E, and it uses a Cas7‐11 effector protein to cleave the target RNA specifically.^137^, ^138^, ^139^

## 3 ANIMAL MODELS USED IN PRECLINICAL STUDIES ON HUMAN DISEASES

Animal models of human diseases are vital in preclinical studies of human diseases. Animal models can accurately simulate the occurrence, development, and clinical manifestations of human diseases and contribute to exploring disease pathogenesis and drug treatment.^8^ A variety of animal models simulating human diseases such as hemoglobinopathies, hematological malignancies, some solid tumors, Leber congenital amaurosis (LCA), Duchenne muscular dystrophy (DMD), diabetes mellitus, and human immunodeficiency virus (HIV) have been developed, and the further exploration of animal models of human diseases will help to improve the preclinical studies of related diseases (Table 3).

**TABLE 3:** Selected animal models used in preclinical studies of human diseases.

Type of disease	Animal models	Applications and characteristics	References
SCD	NOD–SCID–NSG mice; Townes‐SCD mice; NBSGW mice; β‐YAC/CD46 mice; BERK SCD mice; NY1DD mice; SAD mice.	Supporting long‐term xenotransplantation of human stem cells to mimic hemolytic anemia and organ lesions in human SCD to evaluate the therapeutic efficacy of gene editing.	142, 143, 144, 146, 147, 149, 150, 151, 152, 153, 154, 157, 158, 164, 165
β‐Thalassemia	Hbbth1/th1 mice; β654‐ER mice; HBB2 knock‐out rabbit model; NHP model	Mimicking the physiological characteristics of β‐thalassemia for testing gene editing techniques and modifying HbF expression.	172, 173, 175, 176
Hematological tumors	TP53 KO hamster model	Evaluating therapeutic efficacy in preclinical studies of lymphoma and myeloid leukemia.	178
Solid tumors	BPS‐TA mice; BPS‐Cre mice	Simulating common chromosomal deletions in patients with renal cancer.	179
LCA	KCNJ13 mutant mouse model	Exploring the role of causative genes in LCA progression.	181
DMD	ΔEx50 mice	Evaluating the potential of gene editing therapies in DMD by simulating severe muscle dysfunction in humans.	182
Diabetes	GCK‐NFS rabbit model	Mimic diabetes to assess the effectiveness and safety of novel diabetes treatment strategies.	183
HIV‐1	MCM model	Investigating HIV‐1 transmission pathways and treatment strategies.	184

Various mouse models have been developed for use in preclinical studies of SCD (Figure S1).^140^ The primary approach to gene therapy in patients with SCD is to introduce therapeutic gene editing and modification into their HSPCs.^141^ The current gold standard for evaluating the self‐renewal and differentiation‐forming hematopoietic capacity of the HSPCs is via transplantation into immunodeficient nonobese diabetic (NOD)‐severe combined immunodeficiency (SCID)‐gamma (NSG) mice.^142^, ^143^ They support long‐term xenotransplantation of human stem cells, improving the ability to study HSC implantation.^144^ The genetically edited HSPCs were available for transplantation into NSG mice over several months to verify their maintenance level and assess their editing efficiency for research into SCD.^145^ The Townes‐SCD mouse model is produced by mating transgenic mice expressing human HbS (HbSS) with mice with α‐ and β‐globin gene knockouts carrying HBB (HbS) in the form of human HBA with the SCD mutation (E6V).^146^, ^147^ Townes‐SCD mice show severe hemolytic anemia and extensive organ pathological lesions similar to those in patients with SCD. They can be used in drug and gene therapy studies and have been widely used to test the therapeutic effect of gene‐modified HSCs on SCD.^8^, ^148^ Immunodeficient NOD/B6/SCID/IL‐2rγ/Kit (NBSGW) mice facilitate multilineage HSC transplantation with implanted bone marrow, lymph, and RBCs and the continuous transplantation of HSCs.^149^, ^150^ NBSGW mice are a powerful system for studying human erythrocyte production in animal models and have been used to explore the implantation and differentiation potential of gene‐edited HSPCs in mice.^149^, ^150^ Studies have crossed transgenic β‐YAC mice with C57BL/6 mice expressing the human CD46 genomic locus to generate β‐YAC/CD46 mice for in vivo and in vitro HSPC transduction experiments, could be used for SCD and β‐thalassemia studies.^151^, ^152^, ^153^, ^154^ HSPC transduction was performed to detect γ‐globin expression in erythrocytes in healthy β‐YAC/CD46 mice and CD46/Townes mice to investigate whether HSPC transduction could correct SCD.^155^ Since mice lack the fetal β‐like globin gene, other studies have hybridized PAC8.1 mice carrying human β‐globin sites with mice carrying a *Klf1* knockout allele and floxed *Bcl11a* allele to explore the role of the KLF1–BCL11A axis in erythroid maturation and developmental modulation of globin expression.^156^ In addition to the mouse models described above, the Berkley mouse model (BERK) was also used for SCD‐related research.^157^, ^158^ The BERK SCD mice carry disrupted endogenous α‐and β‐globin genes and express HbSS or normal human HbA.^159^, ^160^, ^161^, ^162^ HbSS‐BERK mice have a severe SCD phenotype.^163^ The NY1DD mouse model has a mild SCD phenotype and has linked human α and βs globin transgenes.^164^ The SAD and SAD (beta th/beta +) mouse model has many SCD characteristics, including abnormal hemolysis, vascular occlusion, and microthrombus formation.^165^ These mouse models are stable and reproducible and can be used to study the pathophysiology of SCD and assess therapeutic efficacy.

In preclinical gene editing experiments, using rodents contributes to the feasibility analysis of disease pathogenesis and the implementation of therapeutic methods. However, the mouse model does not fully recapitulate human disease, and long‐term follow‐up studies cannot be conducted to establish the long‐term stability of gene editing and off‐target effects after treatment.^166^ Large animal models such as dogs, pigs, and nonhuman primates (NHPs) are more similar to humans anatomically, immunologically, and lifespan‐wise than small animal models, allowing for longer and more complete studies of clinical drug delivery regimens that should be used in humans.^167^ Studies using large animals for genome‐editing research could bridge the rodent‐clinic gap. The NHP rhesus monkey (*Macaca mulatta*) transplantation model has a life span of 25–45 years.^168^ The rhesus monkey autologous HSCT model can potentially be used to study human hematopoietic system‐related diseases due to its high similarity to humans in genomic profile and hematopoietic and erythropoietic production.^169^ A study using rhesus monkeys evaluated the engraftment and HbF induction potential of erythroid‐specific BCL11A enhancer editing, providing ideas for clinical translation of gene‐edited hematopoietic stem cells (HSCs).^170^ There are also studies using NBSGW mouse and rhesus monkey β‐to‐βs globin conversion models to mimic SCD for genomic editing of HSCs, which provides a research basis for HSC‐targeted gene correction experiments.^171^

In addition to an animal model of β‐thalassemia similar to the SCD animal model, a study used CRISPR–Cas9 to knock down the HBB‐B1 gene in ES cells to construct a mouse model of β‐thalassemia (Hbbth1/th1) in vitro, which was used to explore the molecular basis and therapeutic options for β‐thalassemia.^172^ β^654^‐thalassemia mice containing the βIVS‐2‐654 splice mutation in the human gene mimic the symptoms of moderate to severe β‐thalassemia, and studies were conducted to construct β^654^‐ER mice capable of correctly splicing the β‐thalassemia by gene editing the mutation and splice site using CRISPR/Cas9.^173^ The therapeutic role of HSC transplantation in β^654^‐ER mice was further explored, and the results showed that HSC from gene‐edited mice was able to achieve hematopoietic reconstruction and long‐term hematopoiesis in vivo, and completely restored the phenotype of β‐thalassemia, and the construction of the mouse model provided a reference for the clinical application of gene‐edited HSC.^174^ Rabbits are attractive animal models of human disease with body sizes larger than mice and rats. The study used CRISPR/Cas9 to knock down the HBB2 gene in rabbits to generate a rabbit model of β‐thalassemia, in which heterozygous F1 has the typical features of β‐thalassemia, which is conducive to the study of β‐thalassemia pathogenesis and therapeutic targets.^175^ In a NHP auto transplantation model, CRISPR/Cas9‐edited HSPC had up to 30% transplantation efficiency, efficient and stable activation of HbF in NHP, an editing frequency of more than 25% detected in peripheral blood, and the proportion of erythrocytes expressing HbF was up to 18%. The phenotype of CD34CD90CD45RA and HBG CRISPR/Cas9 gRNA target sites are conserved between NHPs and humans, helping to facilitate the clinical translation of HSPC‐based hemoglobinopathies.^176^ The golden Syrian hamster (Mesocricetus auratus) is also an important model of human disease, and KO of some genes that do not produce good results in mice could present a disease phenotype similar to that in humans in the golden Syrian hamster.^177^ Using CRISPR/cas9, a 1bp insertion at amino acid 311 of the hamster TP53 gene was used to construct a TP53 KO hamster model, in which TP53‐pure mutant hamsters suffer from a variety of cancers, including lymphomas and myeloid leukemia, and TP53‐heterozygous mutants suffer from aggressive acute myeloid leukemia (AML), and could be used as a complementary model to the existing mouse model.^178^

Genetically engineered mouse models are used for the study of single gene action in cancer and are important models for probing disease mechanisms and therapeutic strategies. Studies have used CRISPR–Cas9 to develop triple transgenic mice, BPS‐TA and BPS‐Cre, that induce low numbers of somatic mutations in the tumor suppressor genes Bap1 and Pbrm1 (in addition to Setd2) known to be involved in human ccRCC, mimicking the widespread chromosome 3p deletion common in patients with renal cancer.^179^ It has been demonstrated that a pure nonsense mutation in potassium inwardly‐rectifying channel subfamily J member 13 (KCNJ13) causes patients suffering from LCA.^180^ Research using CRISPR–Cas9 to target the mouse KCNJ13 start codon to generate a mouse model with a nonsense mutation in KCNJ13, confirming the role of KCNJ13 in regulating photoreceptor degeneration in patients with LCA.^181^ One of the most common single‐exon deletions in DMD is a deletion in exon 50 of the antimorphic protein gene, which could be corrected by skipping exon 51, thereby restoring the antimorphic protein open reading frame (ORF). It has been shown that CRISPR/Cas9 deletion of exon 50 was used to generate a DMD mouse model ΔEx50, which exhibited severe muscle dysfunction similar to that of humans, and then CRISPR/Cas9 skipping of exon 51 was utilized to correct the antimyotonic ORF in a mouse model, restoring up to 90% of antimyasthenia gravis protein expression in skeletal muscle and heart of ΔEx50 mice, CRISPR/Cas9 ΔEx50 mice could be utilized in broader preclinical studies of disease progression and treatment of DMD.^182^

Mutations in glucokinase (GCK), which is used for glucose sensing and glucose regulation, induce maturity‐onset diabetes of the young type 2 (MODY‐2), and because GCK KO mice die within 2 weeks of birth and cannot be used for preclinical studies, a study has developed a diabetic rabbit model using CRISPR/Cas9 to produce a nonshift coding mutation (GCK‐NFS) in the GCK gene, which produces hyperglycemia and glucose intolerance typical of MODY‐2 patients, and is a valuable rabbit model for preclinical studies of diabetes mellitus.^183^ Mauritius crab‐eating monkeys (MCM) have a high degree of major histocompatibility complex (MHC) allele sharing and are a valuable model for HIV‐1 research. Since individuals lacking CCR5 have the advantage of being protected from HIV infection, the study utilized CRISPR–Cas9 to edit the CCR5 gene in MCM embryos, introduced a double allelic deletion of the CCR5 gene into MCM embryos, and improved the in vitro fertilizationmethodology to create MCMs with the CCR5 mutation, which could be used in the exploration of HIV therapies.^184^

While the results of animal experiments cannot all be translated into the clinical treatment of human diseases, these animal models of human diseases with different characteristics provide a scientific basis for preclinical studies of human diseases, help to study the mechanisms of disease occurrence and development in depth and promote the clinical translation of gene editing‐based therapeutic strategies.

## 4 GENE THERAPY IN HUMAN DISEASES

In recent years, the US FDA has approved several clinical trials of gene editing technologies in hematological disorders, solid tumors, immune disorders, ophthalmic diseases, and metabolic disorders, foreshadowing the promise of gene therapy for use in the clinical treatment of selected human diseases. In late 2023, the US FDA approved the first CRISPR gene editing therapy, Casgevy, for the treatment of SCD patients with recurrent vascular occlusion crises. Casgevy is the first genome editing therapy to be approved by the US FDA and represents a landmark advancement in the use of gene therapy in human diseases, confirming the promise of gene therapy for clinical Applications. Among the clinical gene therapies that have been approved by the US FDA, the diseases are mainly focused on hemoglobinopathies, hematological malignancies, some solid tumors, LCA, DMD, diabetes mellitus, and HIV, and gene therapy for SCD has been particularly well studied. Below, this article focuses on preclinical and clinical gene therapy research for SCD and outlines gene therapy‐related research for other US FDA‐approved diseases.

### 4.1 Gene editing therapy for SCD

SCD is the most common autosomal recessive genetic disorder, and its severe manifestations and high mortality rate impose a significant global burden.^185^, ^186^ SCD is a classic Mendelian disease that often causes acute and chronic end‐organ damage.^187^, ^188^ Its typical clinical feature is acute pain.^189^ During the disease, complications such as stroke,^190^ kidney disease,^191^ acute lung injury,^192^ myocardial injury,^193^ arrhythmia, and sudden death may also occur.^194^ Moreover, patients with SCD are susceptible to myeloid malignancies and death from diseases such as COVID‐19.^195^, ^196^, ^197^ SCD results from inheriting an abnormal β‐globin allele carrying a sickle mutation.^190^ A point mutation occurs at the sixth position of the Hb beta chain causing glutamate to be replaced by valine (βGlu6Val).^198^ Hb containing this mutated beta chain polymerizes in the red blood cell (RBC) during deoxygenation,^193^ turning the RBC into a sickle shape and causing complex pathophysiology, resulting in hemolytic anemia,^199^ vascular occlusive crisis,^200^ chronic organ injury,^201^ and early death.^202^ Changes in hematological characteristics, such as fetal Hb (HbF) levels, influence SCD.^203^ HbF can blunt the SCD pathophysiology and moderate its clinical course.^204^

**FIGURE 4:**
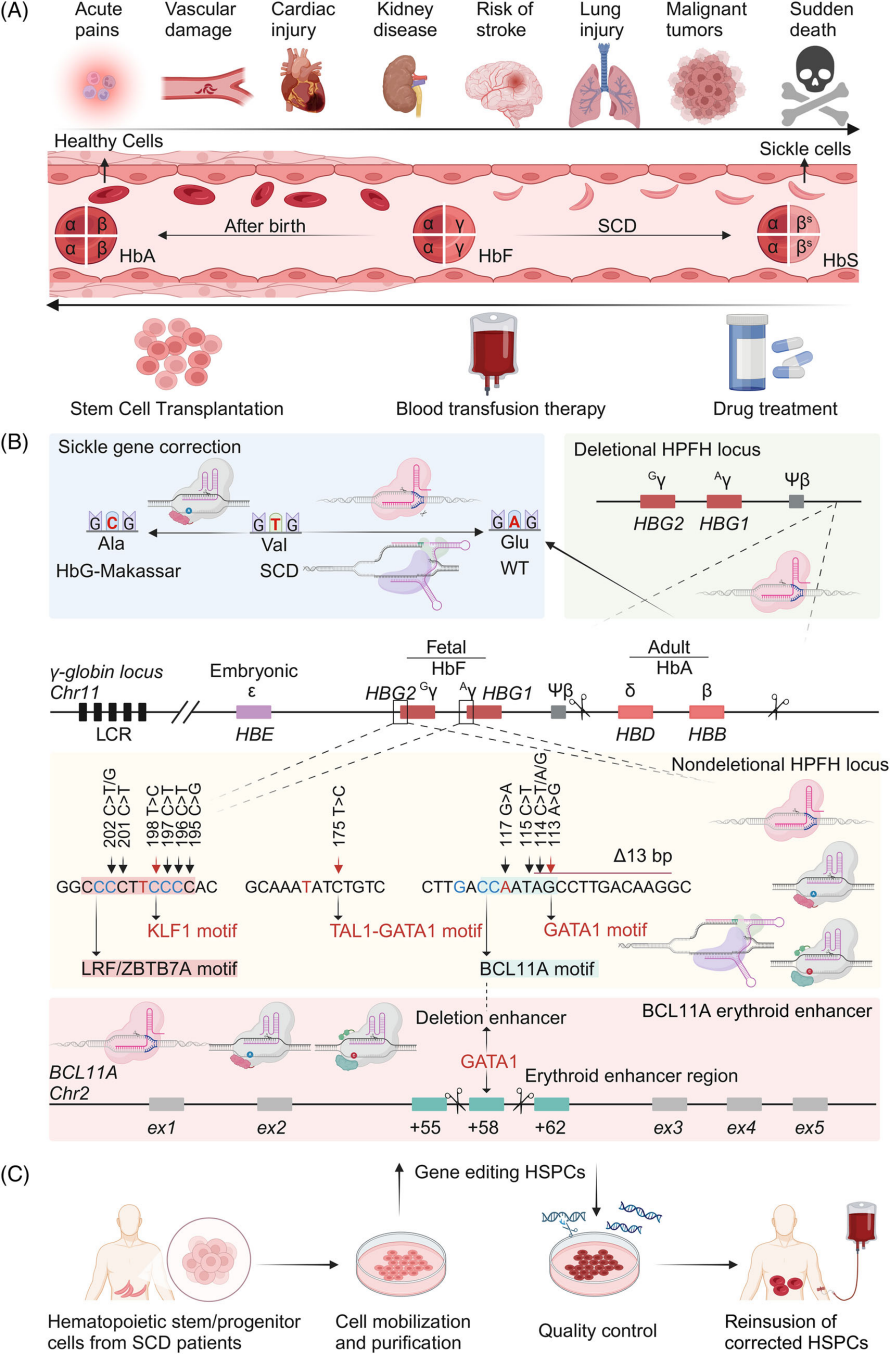
The health risks of SCD to patients and the major SCD gene therapy strategies based on the CRISPR–Cas gene editing technology. (A) Possible diseases in patients with SCD and available therapeutic strategies. The fetal γ‐globin genes (HBG1 and HBG2) are expressed in late gestation, leading to erythrocyte HbF production. Around birth, γ‐globin expression decreases and is replaced by β‐globin, resulting in a shift from HbF to HbA (α2β2) under normal conditions or to HbS (α2βS2) under SCD conditions. (B) Gene therapy strategies for SCD. The blue background indicates the correction of sickle mutations by gene editing to generate wild‐type or benign HbG‐Makassar variants. The green background indicates the deletion of the BCL11A binding site necessary for silencing HbF to introduce the deletion of HPFH. The yellow background indicates interference with the expression or function of the HBG1/HBG2 transcriptional repressors BCL11A or ZBTB7A. Targeting the binding site of ZBTB7A or BCL11A within the promoter or introducing the activators TAL bHLH transcription factor 1 erythroid differentiation factor (TAL1) or KLF1, known HPFH mutations, mimics nondeficient HPFH. The pink background indicates the targeted disruption of the erythroid‐specific BCL11A gene enhancer using gene editing techniques. (C) Brief steps in the gene editing of HSPCs in SCD treatment. HbF, fetal hemoglobin; HbA, adult hemoglobin tetramers; HbS, eliminate sickle hemoglobin; WT, wild‐type; ex, exon. Image created in BioRender.com.

Treatment options for SCD generally include blood transfusion, iron clearance, and medication, which could partially improve the life expectancy of patients with SCD, but these methods do not fundamentally cure SCD (Figure 4A). HSC transplantation (HSCT) is considered the definitive treatment for SCD, offering a potential cure.^205^, ^206^, ^207^ Allogeneic HSCT (allo‐HSCT) has been used as a treatment approach for pediatric and adult patients diagnosed with SCD. Allo‐HSCT has a long‐lasting therapeutic effect in patients but is associated with serious complications.^208^ Moreover, SCD patients requiring allo‐HSCT transplantation often lack suitable donors and are at risk of secondary malignancy after allo‐HSCT transplantation.^209^, ^210^, ^211^ In contrast to allo‐HSCT, autologous HSCs are easy to obtain and have a high rate of transplantation success, but have the potential to lead to disease recurrence.^212^ In preclinical studies of SCD, CRISPR–Cas9‐based gene editing has mainly focused on the in vitro genetic modification of autologous hematopoietic stem/progenitor cells (HSPCs) from patients with SCD, designed to perform a one‐time genetic modification on HSPCs of patient origin, which avoids the requirement for allogeneic healthy donor HSPCs and produces corrected blood cells in the later life of patients with SCD without immunological complications.^141^, ^213^, ^214^ SCD is the first time the CRISPR–Cas system has been used to edit HSCs/HSPCs to treat a patient's disease.^215^ Genomic modification of HSPCs using gene editing technology has shown great clinical promise.^216^ Gene editing introduces therapeutic gene editing and site‐specific gene correction into its HSPCs.^141^, ^217^ The in vitro gene editing of HSPCs is mainly performed in the following ways: (1) The mutated *HBB* gene is repaired either by correctly the mutated SCD‐associated codon from valine (GTG) back to the wild‐type glutamate (GAG) or the naturally occurring benign alanine (GCG) found in the nonsickling HbG‐Makassar variant.^8^, ^122^ (2) Gene therapy for SCD is achieved by interfering with the expression and function of the HBG1/HBG2 transcriptional repressor BCL11 transcription factor A (BCL11A) or zinc finger and BTB structural domain‐containing 7A (ZBTB7A) or by inducing the hereditary persistence of HbF (HPFH), which raises the level of HbF.^218^, ^219^

#### 4.1.1 Induction of HBB gene correction

SCD mutation correction is an effective strategy for preclinical studies of SCD using gene editing technology, which is divided into two methods: one is to directly repair the mutated SCD codon (valine) to the normal codon (glutamate), and the other is to convert the mutated SCD codon to the benign nonsickle G‐Makassar variant (alanine; Figure 4B).^218^

Direct correction or modification of genetic variation is a strategy to cure inherited diseases, preserving elements of regulation that maintain normal gene function and avoiding the adverse consequences that might result from mutating other genes.^220^ Correction of GTG to GAG in SCD patients is a simpler gene therapy approach. Previous studies have used the Cas9 ribonucleoprotein (RNP)/single‐strand oligonucleotide donor (ssODN) to edit the SCD SNP in K562 cells or induce the editing of HDR‐mediated mutations in the SCD *HBB* gene in HSPCs, achieving up to 33% SCD mutation sequence replacement. The differentiation of corrected HSPCs into RBCs produces wild‐type Hb, resulting in a decrease in sickle β‐globin and an increase in adult wild‐type β‐globin and fetal β‐like (γ) globin.^145^ Targeting abbreviations in the *HBB* locus were reduced using eSpCas9‐1.1 and HF1, and an off‐target analysis performed using a web‐based software tool showed minimal off‐target gene activity.^221^ CRISPR–Cas9 and recombinant adeno‐associated virus serotype 6 (rAAV6) targeted editing of HSCs achieved homologous recombination of the *HBB* gene, effectively correcting SCD‐associated amino acid mutations at the *HBB* gene codon 6 (E6V) in HSPCs from multiple patients with SCD, realizing the treatment of patients with SCD.^142^ HiFi Cas9 mediated the correction of high p.E6V mutation levels in SCD‐causing HSPCs of patients with SCD.^24^ The HiFi Cas9 variant achieved more specific gene correction than hSpCas9 in SCD CD34^+^ HSPCs. The off‐target sites were identified using the web‐based simulation mismatch search tool COSMID (CRISPR Off‐target Sites with Mismatches, Insertions, and Deletions) and genome‐wide unbiased mismatch search tool GUIDE‐Seq (Genome‐wide Unbiased Identification of Double‐stranded breaks Enabled by Sequencing).^222^, ^223^ The off‐target effect of HSPC implantation in vivo was found to be very low, proving that the gene editing of HSPCs in patients with SCD was an effective gene therapy approach.^224^ Reversing the sickle phenotype in patients using HSCT requires at least 20% donor myeloid chimerism (i.e., achieving at least 20% of the HSC correction level).^225^ The genomic editing technology potentially corrects SCD mutations in HSCs to simultaneously produce adult Hb tetramers (HbA) and eliminate sickle Hb (HbS). In vitro, HiFi Cas9‐AAV6 has been used to correct *HBB* gene mutations in HSCs from patients with SCD. In vivo, it achieved 20% correction efficiency in human‐derived globin cluster Townes‐SCD mouse models, achieving stable Hb A (HgbA) production and restoring RBC function.^148^ HSC‐targeted genome editing has been simulated in xenografted mice and rhesus monkeys in β‐βs‐globin conversion models, resulting in therapeutic‐level gene correction in mice.^171^ Studies further developed a therapeutic cell product (gcHBB‐SCD) for *HBB* correction in HSPCs using ex vivo Cas9‐RNP and rAAV6 to achieve up to 34% *HBB* allele correction in HSPCs from donor patients with SCD without abnormal hematopoiesis, genotoxicity, or tumorigenicity.^226^ It is safe, effective, and reproducible for initiating phase I/II clinical trials in patients with SCD. Another study found that a high‐fidelity Cas9 (R691A) variant enabled efficient gene editing in HSPCs and better in RNP delivery.^24^

HSPCs are regular cellular targets for gene therapy of hematological diseases, frequently used in most studies. Induced pluripotent stem cells (iPSCs) have also been used to study SCD. iPSCs could be generated from various somatic cells with significant potential in personalized cell therapy.^227^ Correction of pathogenic iPSC mutations can restore function and provide an abundant source of cells for transplantation. Therefore, genome editing of autologous HSPCs and iPSCs mediated by CRISPR–Cas9 is a therapeutic option ideal for treating inherited hematologic diseases.^227^ iPSC banks have been established for patients with SCD with different ethnicities, *HBB* haplotypes, and HbF levels. iPSC technology is an ideal choice for screening drugs against the genetic variants found in the patient population, supporting specific therapies for patients with SCD.^228^ The gene editing of iPSC lines derived from patients with SCD using Cas9 RNP combined with an AAV6 vector achieved a monoallelic editing frequency of up to 94% at the *HBB* locus, greatly improving the clinical applicability of gene editing.^229^ The PE system has realized efficient target editing with few insertions and deletions and no detectable off‐target editing. HEK293T cells homozygous for the SCD‐inducing E6V mutation were treated with PE3 and programmed pegRNA, achieving 26–52% effective correction of the HBB E6V mutation to wild‐type HBB.^122^ Recently, studies have used PE to correct the HBB^S^ allele in HSPCs from patients with SCD into the wild‐type HBB^A^ allele. After transplantation into immunodeficient mice for 17 weeks, the edited HSPCs maintained the HBB^A^ level. It also showed similar hematopoietic differentiation to unedited HSPCs derived from healthy donors, supporting the feasibility of one‐time PE for SCD treatment.^230^

The HbS allele of β‐globin represents the change from GAG (Glu) to GTG (Val) at amino acid six of HBB, and this SNP is converted into GCG (Ala) by A•T‐G•C conversion on the opposite strand using ABE, producing the HBB E6A genotype, the Macassar allele (HbG). There was no pathogenicity in homozygous or heterozygous individuals.^28^ The targeting range of SpCas9 and its engineered variants is mainly limited to G‐base‐containing PAM sequences. Novel SpCas9 variants have been developed to effectively edit the bases of more pathogenic SNPs that are not G‐PAM. Three newly evolved SpCas9 variants have been developed that jointly identify NRNH PAMs (H represents A, C, or T) and convert SCD‐associated SNPs into Makassar mutations by A‐T to G‐C base editing via a CACC PAM. ABE‐NRCH has shown the greatest editing activity with 41 ± 3.8% base conversion.^28^ Base editing strategies using novel PAM variants offer a hopeful treatment for patients with SCD. In SCD, the GAG (Glu) codon encoding β‐globin amino acid 6 mutates to GTG (Val), and the single BE ABE converts the A‐T bp to G‐C in living cells without needing DSBs or donor DNA templates. That is, it converts the pathogenic SCD‐associated codon into GCG (Ala) to produce the rare, occurring naturally, nonpathogenic HBB variant Hb‐Makassar (HBB^G^), where editing more than 20% of HBB^S^–HBB^G^ is sufficient to rescue the SCD phenotype.^8^ Some studies have developed IBEs based on ABE, which edited the pathogenic sickle cell Hb alleles in HSPCs derived from patients with SCD and transformed them into HbG‐Makassar variants, which can potentially treat SCD.^101^ In vitro, editing of human HSPCs has been studied using a customized ABE (ABE8e‐NRCH) by electroporation of ABE8e‐NRCH+sgRNA RNP or ABE8e‐NRCH mRNA with sgRNA (base editing using mRNA or RNP). The SCD‐associated HBB^S^ allele was converted to HBB^G^ with a conversion rate of up to 80%.^231^ The edited HSPCs could be permanently edited when transplanted into immunodeficient mice, with a 68% HBB^G^ frequency at 16 weeks posttransplantation. ABE8e‐NRCH has the smallest nonresting bypass editing in patient CD34^+^ HSPCs. A single autologous treatment eliminates pathogenic HBB^S^, produces benign HBB^G^, and minimizes the DSB adverse consequences, suggesting that in vitro autologous base editing and HSC transplantation are promising methods for treating SCD.^8^

#### 4.1.2 Induced increase in HbF

The γ‐globin and α‐globin subunits pair to form HbF.^232^ HbF passivates the pathophysiology of SCD and moderates the clinical course. Increasing the HbF level is one of the effective treatments to improve the clinical manifestations of SCD.^204^, ^233^ The fetal γ‐globin genes *HBG1* and *HBG2* are expressed in late gestation and induce erythrocyte HbF production. Around birth, *HBG1* and *HBG2* expression is suppressed, γ‐globin levels decrease, and β‐globin levels increase. While HbF is gradually converted to HbA under normal conditions, HbF is converted to HbS in patients with SCD. Therefore, inhibiting the conversion of γ‐globin to β‐globin is a useful strategy for treating SCD.^218^ Gene editing technology can repair β‐globin mutations in the HSCs of patients with SCD, add β‐globin genes with an antisickle effect, activate target genes related to endogenous γ‐globin expression, improve γ‐globin levels, promote normal HbA formation, generate genetic changes to induce HbF expression, improve HbF levels, and obtain long‐term phenotypic correction.^234^ KLF transcription factor 1 (KLF1) is a critical regulatory molecule engaged in the γ‐β‐globin gene conversion process, directly inducing β‐globin gene expression and indirectly suppressing γ‐globin expression. CRISPR–Cas9 can induce the insertion or deletion of the *KLF1* gene in adult erythroid progenitor cells to promote the expression of the γ‐globin gene and increase the HbF level.^235^ In HSPCs derived from patients with primary SCD, CRISPR–Cas9 targeted the deletion or inversion of a genomic region of the HbF silencer between a 13.6 kb δ‐ and γ‐globin gene and a putative gamma‐δ gene. It reactivated HbF synthesis in adult erythrocytes and induced high γ‐globin expression in erythrocytes, which plays an antisickling role in SCD.^236^ CRISPR–Cas9 (Helper‐dependent adenoviral vectors (HDAd)–HBG–CRISPR) was also used to transactivate HSPCs from human β‐globin locus transgenic (β‐YAC) mice in vitro and in vivo, disrupting the repressor binding region in the γ‐globin promoter and converting human β‐globin to γ‐globin in adult mouse erythrocytes. The treatment of SCD was simplified, and no hematological abnormalities were observed, indicating that *HBG1/2* promoter editing is a safe method for treating SCD.^153^ Another study found that CRISPR–Cas9 genome editing of the repressor binding site within the γ‐globin promoter could reactivate endogenous γ‐globin. The gene‐edited HSPCs were transduced into healthy mice and SCD mouse models, significantly increasing γ‐globin levels in the erythrocytes of SCD mice, reaching 30% of the α and βS chains of adult human cells. Their HbF expression level was also increased to that of normal cells, completely correcting the sickle cell phenotype and providing new insights into treating SCD.^155^

Oncogenes BCL11A and ZBTB7A (formerly called lymphoma‐related factor [LRF]) are cis‐regulatory elements (CREs) involved in inhibiting γ‐globin expression, which directly bind separately to fetal β‐globin promoter elements located at −115 and −200, shutting down HbF genes and inhibiting HbF expression early in life.^156^, ^237^, ^238^, ^239^, ^240^ Reversing BCL11A inhibition of γ‐globin expression and reactivating HbF via gene editing represents a promising strategy for SCD therapy (Figure 4B).^241^, ^242^ After editing the GATA binding protein 1 (GATA1) binding site in the BCL11A enhancer +58 using the Cas9 RNP complex to reduce *BCL11A* expression and increase fetal γ‐globin, the HbF level in patients with SCD increased from 13.9 to 47.5%, indicating that the gene editing resulted in long‐lasting HbF induction, and CIRCLE‐seq (Circularization for In vitro Reporting of CLeavage Effects by Sequencing) did not detect off‐target activity.^243^ In addition, a Cas9:sgRNA RNP complex was constructed to target the BCL11A binding motif in the γ‐globin gene promoter in HSPCs, abolishing the BCL11A binding site and increasing the HbF level to a therapeutic level, which is a highly specific and safe treatment strategy for SCD.^244^ Gene editing of erythrocyte‐specific BCL11A enhancers in HSPCs of patients with SCD can also induce HbF expression without detectable toxicity.^244^, ^245^ Due to the similarities between NHPs and humans in genomic mapping and hematopoietic and erythropoietic production, one study evaluated the autologous implantation and induction potential of HSPCs edited with erythrocyte‐specific BCL11A enhancers in four NHPs.^170^ The edited HSPCs lasted for at least 101 weeks after transplantation, produced strong γ‐globin induction during erythropoiesis, provided therapeutic HbF levels in peripheral blood erythrocytes, and had no apparent hematological toxicity or off‐target gene editing, suggesting that BCL11A erythrocyte enhancer genome editing is promising for further clinical transformation. There is frequent cytosine base editing at the BCL11A+58 RBC enhancer. Some studies have used BEs to target the +58 BCL11A RBC enhancer in HSPCs to transform C into T, G, or A, improve the globin chain imbalance, and further achieve efficient multiple editing by jointly abolishing the BCL11A RBC enhancer and correcting the HBB‐28A>G promoter mutation to induce HbF expression and prevent sickle cell formation in RBC progeny.^245^ Base editing can also treat SCD by disrupting the CREs that negatively regulate *HBG1*/*HBG2* expression to increase HbF levels. Previous studies have used ABEmax to insert an A•T to G•C change in the CREs of *BCL11A*, intergenic HBS1‐like translational GTPase (*HBS1L*) to MYB proto‐oncogene, transcription factor (TF) (*MYB*) region, *KLF1*, and β‐like globin genes controlling erythrocyte HbF expression to quantify their impact on γ‐globin gene regulated HbF expression, increases in HbF levels, and provide potential therapeutic insights for SCD.^246^ Studies have developed lentiviral vector (LV)‐based gene addition and CRISP–Cas9 SCD treatments. HSPCs were genetically modified by combining antisickling globin (AS3) in Hb tetramers that disrupted the BCL11A binding site in the *HBG* promoter with CRISPR–Cas9‐mediated disruption or downregulation of BCL11A‐induced γ‐globin reactivation.^247^ Editing of the *HBG* promoter enhanced the healing effect of the AS3 gene addition strategy, increased the total Hb content in β‐globin treated transgenic cells, and induced HbF expression.

In addition to targeting HbF repressors such as BCL11A, another gene editing strategy to treat SCD uses gene editing technology to induce deletion of the β‐globin gene cluster with rare naturally occurring β‐globin mutations in patients with SCD, resulting in HPFH‐related or similar mutations to improve SCD clinical severity.^236^ HPFH is a rare benign disease caused by mutations upstream of the transcriptional start site in *HBG1* and *HBG2*. HPFH induces sustained high HbF expression by reducing the conversion of γ‐globin to β‐globin and disrupting the binding element of BCL11A, a γ‐globin gene repressor (Figure 4B).^248^, ^249^ Some studies used CRISPR–Cas9 to modify the 13‐nt sequence in the *HBG1*/*2* gene promoter of HSPCs to simulate the HPFH‐related mutation. After gene editing, the HbF level in RBCs produced by HSPCs increased, reaching a potentially therapeutic level and providing a theoretical basis for treating SCD with HPFH‐related mutations induced by gene editing.^249^ Some studies have used CRISPR–Cas9 to introduce HPFH‐related point mutations into erythrocytes, disrupt the γ‐globin gene promoter, inhibit BCL11A and ZBTB7A/LRF binding to the γ‐globin gene promoter, induce and enhance γ‐globin gene expression, improve the HbF level, and effectively improve the clinical symptoms of SCD.^219^ Other studies have used CRISPR–Cas9 to edit γ‐globin repressor ZBTB7A/LRF binding sites to simulate HPFH mutations, restore HbF synthesis, and correct SCD phenotypes, suggesting that ZBTB7A/LRF binding sites are effective targets for SCD genome editing therapy.^250^ In addition to the above, TAL1 is an erythroid regulator that acts synergistically with GATA1, enabling T>C at the TAL1‐GATA1 binding site at γ‐bead protein‐175 to mimic HPFH.^251^ Studies have found that deleting the CTCF binding site (CBS) at the 3′ end of the β‐globin locus using CRISPR–Cas9 technology induces HPFH, increases γ‐globin gene expression, and induces HbF expression in HSPCs. Gene editing of the CBS 3′HS1 is a new therapeutic strategy for SCD.^252^ ABE and CBE have been used to introduce −123T>C and −124T>C HPFH‐like mutations within the homologous *HBG1* and *HBG2* promoters to drive γ‐Hb enrichment by creating a KLF1 binding site that increases HbF‐positive cell numbers and overall HbF production.^253^ HPFH is also associated with cascades of deletions within the β‐globin locus downstream of the fetal *HBG2* gene. Using CRISPR to induce mutations that reduce Hb subunit beta (*ΗΒΒ*) promoter activity and increase HbF expression has been studied as an effective treatment for SCD.^254^ There have been studies using ABE (ABE8e) for installing the −113 A>G HPFH mutation in the γ‐globin promoter of healthy CD46/β‐YAC mice with the human β‐globin locus, inducing >60% effective −113 A>G conversion, activating γ‐globin expression, and correcting the erythrocyte phenotype.^154^ No off‐target editing was detected in CIRCLE‐Seq, suggesting that base edit‐induced HPFH mutations have clinical potential in SCD. Some studies have used base editing ABE and CBE to edit the HBG promoter −200 region of HSPCs in patients to induce HPFH‐like mutations, create KLF1 activator binding sites, and induce high levels of HbF expression.^150^ Base editing of the *HBG* promoter avoids insertion, deletion, and large amounts of genome rearrangement and can induce higher γ‐globin levels and save the SCD cell phenotype. GUIDE‐seq found that a small amount of off‐target production did not significantly affect protein expression, suggesting that base editing‐induced HPFH mutation is a safe strategy for SCD treatment.

### 4.2 Gene editing therapy for thalassemia

Thalassemia, similar to SCD, is a common human single‐gene genetic disorder that accounts for more than half of the global disease burden.^255^, ^256^ Thalassemia leads to the production of ineffective RBCs, mainly by affecting the synthesis of the pearl protein chain.^257^, ^258^ The pathogenesis of thalassemia is the disturbance of the equilibrium between α‐like and non‐α‐like (β‐ and γ‐) globin chain production, which is clinically classified into α‐thalassemia and β‐thalassemia, of which β‐thalassemia is the main subtype in gene therapy research.^202^, ^257^, ^259^ The α‐thalassemia and β‐thalassemia are classified as mild and severe depending on the severity. Nontransfusion‐dependent thalassemia is a mild form of thalassemia that is indistinguishable from normal has no complications, and is treated with Luspatercept and Agios Pharmaceuticals.^260^, ^261^, ^262^, ^263^ Transfusion‐dependent β‐thalassemia (TDT) is a severe form of the disease that requires lifelong transfusions to sustain life, and iron overload caused by repeated transfusions leads to dysfunction of organs such as the heart and liver, and iron chelators are needed to control the iron overload.^264^, ^265^, ^266^, ^267^ The impact of unpredictable and severe epidemics, such as pneumococcal pneumonia, may increase the risk of infectious diseases through blood transfusions.^268^ New drugs and gene therapies for TDT patients are in the process of being developed and tested to reduce anemia and the burden of blood transfusions in TDT patients.^269^

In contrast to the fixed‐point mutations in SCD, the genetic variants leading to β‐thalassemia are predominantly pointed mutations or small deletions in the HBB gene, with a few large deletions in large segments.^270^ More than 400 different mutant alleles have been identified as the cause of β‐thalassemia, affecting HBB expression and gene regulation.^271^ Similar to gene therapy for SCD, gene therapy is rapidly emerging as a potentially curative treatment option for β‐thalassemia, and several research protocols are available for both SCD and β‐thalassemia.^245^, ^272^, ^273^, ^274^ There are two major gene therapy strategies for β‐thalassemia. One approach is to directly correct potential mutations in the autologous HSPC HBB gene by gene editing techniques, which overcomes barriers to donor availability, reduces risks such as infection and graft rejection, and compensates for functional deficiencies or defects in the mutant gene.^236^, ^275^, ^276^ Another approach is to regulate the expression of genes such as KLF1 or BCL11A in HSPC by gene editing to increase the amount of HbF.^277^, ^278^ Both approaches are mainly based on the editing and modification of HSPC by CRISPR/Cas9 technology to produce the desired Hb and provide a sustained treatment.^279^ Due to the limited clinical experience with gene editing at present, more clinical data and large‐scale trials are needed to demonstrate that gene editing could be a safe treatment for hemoglobinopathies.^264^ Some of the gene therapy studies for β‐thalassemia are summarized below.

#### 4.2.1 Targeting the HBB gene

Correction of HBB gene mutations in β‐thalassemia using gene editing contributes to the restoration of functional HBB gene expression. One study used CRISPR/Cas9 to achieve targeted insertion of the HBB cDNA in exon 1 of the HBB gene to correct the HBB mutation in human iPSCs derived from β‐thalassemia patients, which allowed the iPSCs to expand and differentiate to generate erythrocytes that could produce corrected HBB protein.^280^ A study applied CRISPR–Cas9 combined with piggyBac transposon to seamlessly correct the HBB mutation in iPSCs derived from β‐thalassemia patients. The gene‐corrected iPSCs did not show off‐target effects and restored the expression of the HBB gene when differentiated into erythrocytes in vitro, suggesting that stem cell‐based gene therapy could be applied to single‐gene disorders.^281^ It has been shown that seamless correction of β‐41/42 deletion mutations in β‐thalassemia patient‐specific iPSCs has been achieved using CRISPR–Cas9 and ssODN with high efficiency and that the corrected cells exhibited minimal mutation loading, no off‐target mutagenesis, and the genetically corrected cells expressed normal β‐globin transcripts when the iPSCs were differentiated into erythrocytes.^282^ Mutations in β‐thalassemia produce aberrant splice sites, disrupting the splicing of the HBB gene and interrupting the alleles of the aberrant splice sites are effective methods to restore gene function. It was shown that CRISPR–Cas9 targeted correction of the IVS‐1‐110 (G>A) mutation site in the HBB locus obtained the wild‐type HBB gene, confirming that gene editing is effective in HBB gene correction.^283^ β^654^‐Thalassemia is the major subtype of β‐thalassemia in China. The study used CRISPR/Cas9 to target the receptor splice sites of IVS2‐654 (C>T) and IVS‐2‐579 to correct aberrant β‐bead proteins for RNA splicing and to improve the clinical symptoms of β^654^ mice, and the gene‐edited heterozygous β^654^ mice showed significantly higher survival rates.^173^ Another study used Cas9 and Cas12a RNPs in β‐thalassemia HSPCs to disrupt aberrant splice sites by acting on the IVS1‐110G>A and IVS2‐654C>T mutations, respectively, and erythrocyte progeny of edited patient HSPCs showed reversal of aberrant splicing and restoration of β‐globin expression, effectively correcting a portion of the TDT genotype.^284^ Studies using multiple neighboring sgRNA‐guided CRISPR–Cas9/RNP editing of HSPC were able to induce deletions of large segments, directly restoring the damage to HBB gene function caused by the IVS2‐654 (C>T) mutation without off‐target effects, making it a highly effective and safe treatment for β‐thalassemia.^285^ In a study, PE3 was used to correct the IVS‐II‐654 mutation (C>T) in a mouse model of β‐thalassemia, and components of PE3 were microinjected into the fertilized eggs of the mice, and gene‐corrected mice with an editing efficiency of 14.29% were successfully obtained, and editing of PE3 restored the normal splicing of β‐globin protein mRNA, which resulted in curative treatment of the mice, and off‐target analyses confirmed the genomic safety of PE3 editing.^286^ One additional study used CRISPR–Cas9 to correct the β^039^ mutation, which resulted in a significant increase in the efficiency of production and accumulation of β‐cadherin and HbA. β‐Thalassemia is associated with the number of inherited α‐globin genes, and when the β‐globin chain is absent, free α‐cadherin deposition disrupts the cellular membranes, generating ineffective erythrocytes and hemolysis. It has been shown that induction of HBA2 deletion in human HSPC using CRISPR/Cas9 can reconstruct α‐thalassemia traits downregulate α‐globin expression, and subsequently improve β0‐thalassemia phenotypes by targeting the endogenous HBA promoter to combine the deletion of HBA2 with the gene replacement of HBB to increase β‐globin gene expression.^287^ The HBB‐28 (A>G) mutation is one of the most common mutations in β‐thalassemia patients in China and Southeast Asia. A study has used BE to precisely correct the HBB‐28 (A>G) mutation in primary cells of patients, with a gene correction efficiency of more than 23.0% in nuclear‐transplanted embryos, and a BE variant with a narrow deamidation window could precisely facilitate HBB – 28 loci, confirming the promise of base editing systems for the curative treatment of human somatic and embryonic genetic diseases.^288^ There is a study using CRISPR/sgRNA‐RNP complexes with rAAV6 donor‐mediated HDR to edit patient‐derived HSCs, to perform in situ gene correction for different HBB mutations in HSCs, to restore β‐globin content in differentiated erythrocytes, and, the edited HSCs have the ability to be transplanted into the bone marrow of immunodeficient NOD–scid–IL2Rg−/− mouse bone marrow, providing an effective and safe method for the treatment of β‐thalassemia patients by HBB locus correction.^289^ HbE β‐thalassemia accounts for approximately 50% of all types of β‐thalassemia, and severe β‐thalassemia is caused when there is a point mutation in the codon 26 allele of the human HBB gene (GAG→AAG, E26K), and any mutation at the other locus of the allele. The study used the CRISPR/Cas9 system to correct the HBB gene in iPSCs from HbE/β‐thalassemia patients, and the corrected iPSCs could be differentiated into HSCs for autologous transplantation in HbE/β‐thalassemia patients.^290^ Another study used the ABE base editing strategy to correct HbE mutations to wild‐type or Hb E26G with a normal phenotype of the Hb Aubenas variant, achieving editing efficiencies of up to 90% in primary human CD34+ cells and analyzing the off‐target effects using CIRCLE‐seq, confirming the base editing strategy as a therapeutic strategy for HbE β‐thalassemia.^291^

#### 4.2.2 Induced increase in HbF

In addition to the correction of gene mutations, inducing an increase in HbF expression is an effective strategy for the treatment of β‐thalassemia.^277^ Restoration of HbF expression has a positive therapeutic effect in most TDT patients compared with repair of the HBB gene.^292^ Consistent with SCD, HPFH in β‐thalassemia likewise improves the clinical phenotype of patients and is a promising treatment for β‐thalassemia.^293^ After disruption of the BCL11A binding site in the HBG1/HBG2 promoter, the introduction of the natural HPFH mutations ‐113A>G, ‐114C>T, ‐1175T>>C, ‐195C>G, and ‐198T>C into HSPC using CRISPR–Cas9/AAV6 significantly increased HbF expression.^294^ In addition to BCL11A, regulators of HbF such as KLF‐1, ZBTB7A/LRF, SOX6, ZNF410, and RBM12 expanded the range of possible genome editing targets.^295^, ^296^, ^297^ The study used a transforming BE (tBE) to directly disrupt the BCL11A binding motif in the HBG1/2 promoter, which could produce durable therapeutic editing in HSPC from β^0^/β^0^ thalassemia patients, significantly enhancing the level of γ‐globin, increasing the level of HbF expression, and without detectable off‐targeting.^298^ Transplantation of tBE‐edited HSPC into immunodeficient mice was detected 4 months later to reconstitute the hematopoietic system in the mice. Both in vivo and in vitro experiments confirmed that tBE editing of BCL11A binding motif induced HbF levels superior to ABE8e and hA3A‐BE3 editing of BCL11A enhancers, and was a highly efficient strategy to reactivate HbF in patients’ HSC. There is a study that used CRISPR–Cas9 to disrupt a 13.6 kb genomic region containing the δ‐ and β‐globin genes as well as a silencing factor for HbF between the γ‐δ genes, which increased γ‐globin expression while inducing activation of HbF, with beneficial effects on the treatment of β‐thalassemia.^236^ It has been shown that deletion of a 200 bp genomic region encompassing the GATAA motif within the BCL11A enhancer of the K562 cell line using CRISPR–Cas9 induced hereditary persistence of HPFH, which resulted in increased HbF expression.^299^ The study used CRISPR–Cas9 to correct gene mutations in erythroid precursor cells from homozygous β039 thalassemia patients, inducing accumulation of the β‐globin gene and efficient production of HbA without genomic toxicity.^300^ It was further shown that HbA and HbF can be induced using rapamycin to induce ab initio expression of HbA and HbF at the same time as CRISPR–Cas9 editing of the β‐globin gene, inducing an increase in HbF.^301^ In order to investigate the difference in the effect of a single gene editing method versus multilocus editing, the study used CRISPR–Cas9 to simultaneously edit both cis (HBG‐promoter binding site) and trans (BCL11A enhancer) target sites and maintained the same high editing efficiency in NBSGW mice as in CD34 cells.^302^ The study optimized the vector capable of editing the HDAd5/35 CRISPR of HSPC to the adenoviral vector Ad‐dualCRISPR that could be used for dual genome editing to further increase the level of HbF reactivation in β^0^/β^0^‐thalassemia mice, expanding the therapeutic window of gene editing to provide a safe and effective therapeutic strategy for phenotypically severe patients. In addition to gene editing using CRISPR–Cas9, a study developed a near PAM‐free ABE variant, ABE8e‐SpRY, which uses ABE8e‐SpRY RNP to correct the HbE (CD 26, G>A) and IVS II‐654 (C>T) mutations in patient‐derived CD34 HSPC, disrupting the BCL11A enhancer +58 site, upregulates the expression of γ‐globin in the BCL11A enhancer or HBG promoter, mimics the HPFH mutation, induces HbF levels at or above the clinical threshold, produces durable therapeutic editing in human HSC and confirms that the editing has a safety profile using the Cas‐OFFinder tool.^303^ There were also studies comparing five strategies using CRISPR–Cas9 or ABE in HSPC, which showed that base‐edited ‐175A>G erythrocyte colonies expressed 81±7% of HbF, whereas unedited controls expressed 17±11% of HbF and CRISPR–Cas9 induced lower levels of HbF than base editing.^304^ When HSPC was transplanted into mice, base editing induced ‐175A>G to produce HbF equally better than CRISPR–Cas9, and the study demonstrated better therapeutic efficacy of base editing compared with CRISPR–Cas9. A study using CRISPR/Cas9 and BE to edit HSPC derived from β‐thalassemia patients achieved efficient editing of the HBG promoter, which resulted in activation of γ‐globin and a significant enhancement of HbF expression levels.^305^ Studies have utilized the HDAd5/35++ vector to deliver ABE, target editing of the BCL11A enhancer, or induce HPFH mutations in the HBG1/2 promoter, to efficiently achieve target base conversion and reactivate γ‐globin production.^306^ It was shown that the ratio of γ‐globin to erythrocytes in the peripheral blood of the ABE vector targeting the ‐113A>G HPFH mutation (HDAd–ABE–sgHBG‐2) was greater than 40% with no detectable off‐target editing, and the rate of HBG1/2 deletion after ABE editing was much lower than that induced by CRISPR/Cas9 in the β‐YAC mouse model, suggesting that the HDAd‐vector delivery of ABE is a promising strategy for the treatment of β‐hemoglobinopathies.

### 4.3 Gene editing therapy for hematological tumors

Diseases of the hematological system mainly include hereditary diseases and cancers, leukemia, myeloma, and lymphoma belong to the malignant cancers of the hematological system, which are malignant diseases originating from the lymphohematopoietic system and may involve all systems and organs of the body.^307^, ^308^ Hematological malignant tumors are highly heterogeneous, with complex manifestations and diverse phenotypes, and are difficult to diagnose in the early stages of the disease and difficult to cure, causing great damage to human health. Chemotherapy, radiotherapy, targeted drug therapy, immunotherapy, and HSCT are the current therapeutic strategies for hematological malignancies.^309^ However, each therapy is only effective for some malignant hematological diseases and some patients fail due to relapse and drug resistance, therefore, precision medicine and individualized treatment for patients with hematological malignancies are still the focus of research.^310^

#### 4.3.1 Acute myeloid leukemia

AML is an aggressive disease with defects in normal hemopoiesis.^311^ Gene editing technology can be applied to genome‐wide screening of AML, which can help to identify targets for differential therapy in AML.^312^ One study established a CRISPR screening method using an in situ xenograft model and confirmed that SLC5A3, which is associated with AML cell metabolism, and MARCH5, a key gene for apoptosis, are candidate targets for AML treatment.^313^ Double‐negative T cells (DNT) are able to kill most AML patients’ primitive cells, but about 30% of AML patients’ primitive cells are resistant to the cytotoxicity of DNT. Studies using CRISPR/Cas9 screening to identify genes in AML cells that are susceptible to DNT treatment have shown that inactivation of CD64 leads to resistance in AML and that the expression level of CD46 is associated with the AML cell susceptibility to DNT correlated.^314^ In addition to CRISPR screening for AML, gene editing technology has also enabled the treatment of AML by editing CAR‐T cells. CAR‐T cell therapy has an important role in hematological tumor therapy, and one study used CRISPR/Cas9‐RNP to knock down CD45 in CAR‐T cells, which after long‐term cultivation in vitro still had efficient therapeutic effects.^315^ In T cells and CAR‐T cells from AML patients, the expression of activated CD69 and HLA‐DR as well as the markers of exhaustion PD1 and LAG3 was increased, leading to a decrease in antitumor efficacy.^316^ Study has combined CRISPR/Cas9 with the viral‐free gene transfer strategy of the Sleeping Beauty transposon to edit CAR‐T cells from patients, generating HLA‐I^KO^/TCR^KO^ CAR‐T cells with HLA‐I and TCR complexes removed, with a high degree of specificity and safety of gene editing, which has enhanced the therapeutic efficacy of CAR‐T cells in AML and is a promising option for the treatment of AML CAR‐T cell therapy is limited by the lack of cancer‐restrictive surface markers, and CAR‐T targeting of CD33, a classical marker in AML, destroys normal myeloid cells, the study used CRISPR/Cas9 to knockdown CD33 in normal HSPC, and achieved long‐term implantation and differentiation in immunodeficient mice and rhesus monkeys, and the xenografts of CD33 KO HSPC with CD33 CAR T constitutes a functional hematopoietic system that can specifically target AML and enable treatment of AML without myelotoxicity, and the findings confirm that on‐target and off‐tumor toxicity could be avoided by genetically engineering the host, providing an important reference for selective tumor targeting in cancer immunotherapy.^317^ According to a study using a CD276‐CAR construct to transduce natural killer (NK)‐92 cells and CRISPR–Cas9 to knock down three different inhibitory checkpoints (CBLB, NKG2A, and TIGIT) in NK cells, triple‐knockdown CD276‐CAR‐NK‐92 cells showed a significant enhancement of toxicity against leukemia cells, and are a promising candidate for the treatment of AML and B cell acute lymphoblastic leukemia.^318^

#### 4.3.2 Multiple myeloma

As a malignant tumor of the hematological system, multiple myeloma (MM) is diagnosed in approximately 588,161 people worldwide each year. In MM, there is an abnormal growth of clonal plasma cells in the bone marrow, which may cause symptoms such as kidney damage, osteolytic bone disease, and anemia.^319^ Moreover, immune dysfunction in patients with MM increases the risk of infectious diseases, and currently, there is still no curative treatment option for MM, only a prolongation of the patient's survival.^320^ Consistent with its role in AML, CRISPR gene editing technology is used for genome‐wide screening of MM therapeutic targets and editing of immune cells in vitro. As existing therapeutic options do not target specific oncogenic mutations in MM, one study used CRISPR for genomic analysis of 19 MM cell lines and hundreds of normal cell lines to systematically characterize the molecular‐dependent preferences of MM and identify 116 important genes affecting MM, which provide valuable targets for targeted therapy in MM.^321^ The clinical use of new drugs has improved the treatment of MM, but most patients end up relapsing due to drug resistance, and the relationship between patient‐specific mutations and sensitivity to drugs has not been explored. The study performed whole exome sequencing of patient samples and CRISPR–Cas9 resistance screening for relapse‐specific mutations against commonly used drugs such as dexamethasone and identified 15 functional mutations that were resistant to different drugs, and the study of resistance due to specific genetic alterations could help to personalize the treatment of MM patients.^322^ Unlike CAR‐T cell therapy, T‐cell receptors (TCRs) recognize antigens of intra‐ and extracellular origin, expanding the scope of targeted therapy. Transfer of tumor antigen‐specific TCR genes into T cells is the more popular therapeutic option, and T‐cell therapies generating high‐frequency transgenic TCRs (tgTCRs) have improved TCR gene therapy. Studies using CRISPR/Cas9 to simultaneously edit the TRAC or TRBC motifs have achieved KO of endogenous TCRαβ, elevated tgTCR expression, improved recognition of MM tumor targets, and effectively prolonged treatment duration, making it an enhanced and highly effective T cell therapy.^323^ NK cells are cytotoxic, and an increase in the number of NK cells improves patient survival in MM therapy, but NK cell function is impaired due to up‐regulation of inhibitory receptors. The study used CRISPR–Cas9 to knock down the killer cell lectin‐like receptor C1 (KLRC1) motif in the immune checkpoint NKG2A in primary NK cells. The gene‐edited NK cells showed significantly increased toxicity to some MM cells and could still produce NK cell‐specific cytokines, the disruption of immune checkpoints in primary NK cells has the potential to be overcome in clinical treatment immune checkpoint suppression, overcome tumor immune evasion and improve therapeutic effects on MM.^324^

#### 4.3.3 Lymphomas

Lymphomas are divided into three main groups: B‐cell lymphomas, NK/T‐cell lymphomas (NKTL), and Hodgkin's lymphomas.^325^ Diffuse large B‐cell lymphoma (LBCL) accounts for approximately 30% of non‐Hodgkin's lymphomas, and patients have symptoms such as progressive lymph node enlargement; more than half of the patients can be cured by R‐CHOP immunochemotherapeutic agents such as rituximab and adriamycin, but the prognosis of patients who are not cured is poor, and new therapeutic strategies need to be developed.^326^ NKTL is an Epstein–Barr virus (EBV)‐associated non‐Hodgkin's lymphoma, lymphoma cells are transformed from NK cells or T cells, EBV infection and cytogenetic alterations are the main factors in the development of NKTL, and most patients are treated with radiation therapy, a few patients with extensive disease are treated with chemotherapy, and immunotherapy and hematopoietic cell transplantation treatments are still in the early stages of exploration.^327^ Classical Hodgkin's lymphoma is a highly curable cancer and patients are usually treated with a combination of first‐line chemotherapy and radiotherapy, but some patients are not cured and die due to the toxic effects of the treatment.^328^ CRISPR/Cas9‐based genome editing offers clinical therapeutic potential for immunotherapies such as TCR, CAR T cells, and over‐the‐counter cell transfer (ACT).^329^ CAR T‐cell therapies are primarily designed to target diseases individually, and one study used ABE base editing to install a function‐preserving mutation that protects CAR T cells and healthy hematopoietic cells from the toxic effects of CD45 targeting, to develop universal CAR T‐cell therapies targeting panleukopoietic CD45 markers for AML, B‐cell lymphoma and acute T‐cell leukemia.^330^ Another study used CRISPR–Cas9 to generate gene‐specific targeted nonviral CAR‐T cells and inserted an anti‐CD19 CAR cassette into the AAVS1 safety motif to develop innovative anti‐CD19 CAR‐T cells integrated with PD1. This two‐in‐one approach was therapeutic for patients with relapsed/refractory aggressive B‐cell non‐Hodgkin's lymphoma, with a remission rate of 87.5% in eight patients, confirming the safety and efficacy of nonviral, gene‐specific integration of CAR‐T cells in oncology therapy.^331^ Super enhancers (SEs) could drive key oncogenes in malignant tumors. The study analyzed primary tumor samples of SEs NKTL and found that TOX2 was aberrantly overexpressed in NKTL. Subsequently, the regulation of SE oncogenes by TFs was explored using techniques such as CRISPR–dCas9, assessed the effect of TOX2 on the degree of malignancy of NKTL in vitro and in vivo, and demonstrated that targeting TOX2 may be an important therapeutic intervention for NKTL patients.^332^ Brentuximab vedotin (BV), a drug‐coupled anti‐CD30 antibody, is an effective agent in refractory/recurrent Hodgkin's lymphoma, but many patients end up developing resistance to BV, a study established a CRISPR library screening platform for BV‐sensitive gene identification in Hodgkin lymphoma for this purpose, and the results showed that ubiquitin editing enzyme A20 was negatively correlated with the activity of NF‐κB, and that NF‐κB could affect the sensitivity to BV treatment by regulating ABCB1 expression; therefore, targeting and regulating the activity of NF‐κB could synergistically modulate the sensitivity of patients to BV.^333^ The above studies suggest that CRISPR gene editing technology plays an important role in both gene therapy of diseases and the exploration of drug resistance mechanisms.

### 4.4 Gene editing therapies for solid tumors

CRISPR gene editing technology offers additional therapeutic strategies for human diseases, and gene therapy has been progressively applied to a variety of human diseases, including solid tumors.^334^, ^335^ The CRISPR system is widely used in preclinical studies of cancer, and the major solid tumors for which the US FDA has approved the use of gene therapy for clinical trials are gastrointestinal (GI) cancers, metastatic non‐small‐cell lung cancer (NSCLC), and recurrent or refractory solid tumors such as clear‐cell renal cell carcinoma (RCC), cervical carcinoma, esophageal cancer, pancreatic cancer, and malignant pleural mesothelioma. Cancer treatments are mainly surgery, chemotherapy, and radiotherapy, and gene therapy generally knocks out or downregulates target genes in cancer cells that are important in the regulation of the cancer process, resulting in a therapeutic effect.^336^ Drug resistance is an important cause of treatment failure in patients, and screening of drug‐resistant and drug‐sensitive genes using the CRISPR system can help to reverse drug resistance in patients.^337^, ^338^ CRISPR screening can also be used to identify immune escape genes and immune‐suppressive checkpoints in cancers, revealing the mechanism of immune escape from cancer.^339^ With the rise of CAR T‐cell therapy, CRISPR–Cas9‐based gene editing technology has been widely used to edit immune cells, such as CAR T‐cells, to provide customized or universal immunotherapy for tumor patients.^340^ GI cancers occur in the GI tract and digestive organs and are influenced by diet, alcohol consumption, geographic factors, and genetic factors.^341^ Studies have shown that MEK inhibition (MEKi) enhances the antitumor capacity of patients and can immunomodulate tumor cells and tumor‐infiltrating lymphocytes (TILs), and studies have utilized the CRISPR/Cas system‐mediated MEK1 KO to mimic tumor‐specific MEKi, and pharmacological MEKi using cobimetinib in colorectal cancer models, thereby distinguishing MEKi mediated and dependent mechanisms of immunomodulation. MEK1 KO tumors displayed a growth‐impaired phenotype with T‐cell activation, and pharmacological MEKi reproduced tumor‐intrinsic effects but compromised T‐cell activation, confirming that T‐cell agonist therapy maximizes the beneficial effects of MEKi in counteracting the immune response to tumors.^342^ NSCLC accounts for approximately 85% of lung cancers and is the most common type of cancer‐related death, with molecularly targeted and immunotherapies improving the prognosis of patients, but patients with advanced disease can become resistant to existing treatment regimens.^343^ In T‐cell therapy, editing of immune checkpoint genes using CRISPR/Cas9 can improve efficacy, and studies using CRISPR/Cas9 to KO PD‐1 in T cells and subsequent infusion of gene‐edited T cells into patients have demonstrated safety and feasibility in patients with advanced NSCLC who have failed multiple lines of therapy, with patients being able to detect TCRs in 4 weeks cloning, confirming the feasibility of gene editing of T cells for clinical application.^344^ Approximately 70% of RCC patients have clear RCC (ccRCC), and some ccRCC patients deteriorate due to metastasis of the tumor leading to death.^345^ Multitargeted tyrosine kinase inhibitors (TKIs) are used for the treatment of patients with advanced ccRCC, but TKIs are not therapeutically effective in many patients, and CRISPR/Cas9 high‐throughput loss‐of‐function (LOF) screening can identify and target cytokines involved in resistance to anticancer therapy. The study used CRISPR/Cas9 LOF screening for the identification of cytokines involved in sunitinib resistance and identified farnesyltransferase as an important contributor to the induction of resistance to sunitinib in patients, and cellular and animal experiments demonstrated that the combination of sunitinib with the farnesyltransferase inhibitor lonafarnib was effective in enhancing the antitumor efficacy of sunitinib in vitro and in vivo.^346^

### 4.5 Gene editing therapy for inherited eye diseases

LCA belongs to the hereditary retinal dystrophy, which is a heterogeneous autosomal recessive disorder that severely affects the patient's visual acuity and is a major cause of childhood blindness.^347^ LCA is mainly caused by gene mutations, among which a deep mutation in intron 26 of the CEP290 gene (c.2991+1655A>G, IVS26 mutation) in LCA10 patients is the most common causative mutation in LCA, which leads to incorrect splicing and early generation of the stop codon affecting protein expression.^348^ In response to the splicing mutation in LCA10, a study used the dual AAV system to deliver CRISPR/Cas9 to target the deletion of the intronic fragment of the CEP290 gene containing the IVS26 mutation in the mouse retina in order to rescue the expression of CEP290, and then experiments were performed in mice, confirming the efficacy of SpCas9 gene editing. The study further developed a self‐limiting CRISPR/Cas9 to limit the duration of SpCas9 expression in vivo to avoid host immune response to SpCas9 affecting the safety of the treatment.^349^ One study used CRISPR–Cas9 to correct a nonsense mutation in the RPE65 gene in a mouse LCA model of type 12 (rd12) retinal degeneration, resulting in homology‐directed repair of RPE65 in mouse retinal pigment epithelial tissues, and achieving therapeutic correction in RPE65 mutations.^350^ In another study, correction of the C to T nonsense mutation (p.R44X) in the RPE65 gene of the rd12 mouse model using the ABE base editor and injection of AAV‐ABE under the retina of mice was able to successfully correct the mutation in RPE65 and restore its light‐induced electrical response, confirming the therapeutic potential of gene editing to correct the disease‐causing mutation in LCA.^351^

### 4.6 Gene editing therapy for DMD

DMD is a severe progressive monogenic muscular dystrophy disorder caused by mutations in the gene encoding antimyasthenic proteins and is the most common inherited myopathy in children.^352^ Mutations in the DMD gene are predominantly single or multiple exon deletions that disrupt the ORF, introduce termination codons early, and produce nonfunctional truncated antimyasthenic proteins, resulting in a severe muscle degenerative phenotype.^353^ Patients lose the ability to walk, develop respiratory insufficiency, and heart failure, and die by the third decade of life.^354^ Therapeutic strategies in preclinical studies of DMD have focused on the restoration of antimorphic protein function.^355^ Studies have used CRISPR/Cas9 to target mutations in exons 45–55, introducing shifts or deleting one or more exons within the exon to restore the ORF of DMD proteins, which corrects 62% of DMD mutations and effectively restores DMD protein expression.^356^ Gene editing has been applied to correct DMD mutations in human adult myocytes and patient‐derived iPSC lines. Dilated cardiomyopathy is one of the lethal causes of DMD, and studies have used CRISPR Cpf1 to edit DMD mutations in patient‐derived iPSCs as well as mouse models to restore antimorphic protein expression by skipping out‐of‐frame DMD exons or correcting nonsense mutations.^357^ ABE could correct nonsense point mutations in mouse models of DMD, and one study used CRISPR–Cas9 to construct DMD human iPSC cells with a large deletion in exons 48–54 (ΔE48–54), which disrupted myotonic dystrophin expression in cardiomyocytes derived from DMD human iPSCs, and subsequently, the study used ABE to convert AG to GG (35.9 ± 5.7%), skipping exon 55, restored myotonic dystrophin expression, suggesting that ABE‐mediated exon skipping has the potential to be used as a gene therapy for DMD.^358^ One study used ABE to edit the splice donor site of the myotonic dystrophy gene to skip the DMD exon 51 deletion mutation (∆Ex51), and PE was used to reconstitute the myotonic dystrophy protein ORF, thereby restoring myotonic dystrophy gene expression.^359^ Delivery of gene editing systems is the main technical bottleneck in gene therapy for DMD.AAV vectors could be used for in vivo delivery, but AAV vectors may induce severe adverse effects in DMD patients. One study constructed a lipid nanoparticle (LNP) system that could deliver the gene editing system into skeletal muscle by repeated intramuscular injections, which induced stable genomic exon jumps and restored antimorphic proteins in a mouse model of DMD with humanized exon sequences, and is a promising vector for DMD therapy.^360^ As the dCas13 protein is large and difficult to apply for AAV delivery, a study reported a smaller RNA BE (ceRBE), which replaced the dCas13 protein with the EcCas6e protein, was able to achieve A to I and C to U base editing with low transcriptome off‐targeting, and in humanized DMD mice, AAV delivery of ceRBE could efficiently repair the DMD Q1392X mutation (68.3 ± 10.1%), confirming the potential application of ceRBE in the treatment of genetic diseases.^361^

### 4.7 Gene editing therapy for diabetes

Diabetes mellitus is a heterogeneous metabolic disorder with abnormally elevated blood glucose concentrations, and diabetic patients typically exhibit symptoms such as polyuria, thirst, and easy fatigue.^362^ Diabetes mellitus is mainly classified into type 1 diabetes (T1D), type 2 diabetes (T2D), gestational diabetes, and diabetes from other causes, with T1D and T2D being the two most common subtypes.^363^ T1D is a chronic autoimmune disease caused mainly by an absolute deficiency of insulin leading to autoimmune pancreatic β‐cell destruction.^364^, ^365^ Insulin resistance in T2D may lead to hyperglycemia with progressive loss of β‐cell function and gradual disappearance of β‐cell insulin secretion, and obesity resulting from a poor lifestyle with a sub‐healthy diet increases the chance of developing diabetes.^366^, ^367^ As a global health problem, most diabetic patients are treated with insulin.^368^ Some patients with diabetes diagnosed early in the disease may be able to achieve curative treatment through diet.^369^ Due to the autoimmune destruction of pancreatic β‐cells in T1D, research has attempted to develop cell replacement therapies to differentiate iPSC into β‐cells for transplantation, but there is an urgent need to optimize the β‐cells so that they could resist autoimmune killing effects due to autoimmune destruction of the transplanted cells. A study performed a CRISPR genetic screen in a mouse model of T1D and confirmed that deletion of RNLS, a candidate gene for genome‐wide association studies in T1D, enables β‐cells to resist killing by autoimmunity and that RNLS is a potential therapeutic target to avoid β‐cell loss in T1D.^370^ It was further shown that the US FDA‐approved drug pargyline, a potential inhibitor of RNLS, could protect transplanted β‐cells in T1D mice and thus be therapeutic for diabetic patients. As the genetic signaling of T2D mainly affects islet cell dysfunction, the study used a genome‐wide CRISPR screen on human pancreatic β‐cell lines to screen for genes regulating β‐cell function, which showed that CALCOCO2 is an important regulator of β‐cell function.^371^ Insulin‐secreting β‐cells (SC‐β) differentiated from iPSCs of diabetic patients could be used as autologous cells in diabetic cell replacement therapy, but SC‐β cells are less functional, and a study has corrected for the diabetogenic pathological variant of Wolfram syndrome 1 (WFS1) in iPSCs of patients with Wolfram syndrome (WS) using CRISPR/Cas9. The corrected WS SC‐β cells had increased insulin secretion, could be robustly differentiated, and transplanted into mice with reversed streptozotocin‐induced diabetes, suggesting that correction of patient‐derived iPSCs using CRISPR/Cas9 may provide cells with therapeutic utility in diabetic cell replacement therapy.^372^

### 4.8 Gene editing therapy for HIV‐1 infection

Acquired immunodeficiency syndrome, caused by HIV infection, is a global health burden. HIV‐1 is the most common subtype of HIV infection and is a complex refractory immune disease with a high mortality rate.^373^ HIV‐1 infection invades the human immune system, and infected individuals often end up dying due to comorbidities of severe infections and malignancies.^374^ HIV infection greatly increases the risk of cancer in patients, and lymphoma is the most commonly developed malignancy in HIV patients.^375^ Protective vaccines and curative treatments have not yet been developed for clinical use. Treatment of HIV patients focuses on maximum and sustained suppression of viral replication in the body, re‐establishing the patient's immune function, and working synergistically with the treatment of other opportunistic infections or cancers to achieve control and salvage of disease progression. Antiretroviral therapy (ART) can inhibit HIV replication, but it requires antiviral drugs and HIV could integrate genes into human chromosomes to evade drugs and the immune system.^376^ HIV infection of cells of the host immune system integrates its genetic material onto human cells for a short period, forming a stable, latent state viral reservoir, ART is difficult to absorb by tissues carrying the latent virus, and the virus may rebound weeks after cessation of treatment.^377^ Elimination of viral reservoirs with integrated replication capacity in host cell DNA using CRISPR–Cas9 gene editing is a promising strategy for achieving a functional cure for HIV.^378^, ^379^ The complete elimination of HIV‐1 has become a major point of research to keep the virus from recurring. Studies have produced long‐acting slow‐release ART (LASER ART) to reduce the frequency of ART administration, and given that LASER ART is unable to eradicate all of the viruses in the body, studies have delivered CRISPR–Cas9 using AAV9, which specifically excises integrated HIV‐1 proviral DNA in the host genome and sequential treatment of humanized HIV‐1‐infected mice using LASER ART and CRISPR–Cas9. It was found that levels of viral and integrated DNA were undetectable in a subset of mice, confirming that this sequential treatment is capable of removing the virus from latent infectious reservoirs and has the potential to achieve complete elimination of replication‐competent HIV‐1 in human‐infected individuals.^380^ A study combining gene therapy with long‐acting antiretroviral drug therapy has used CRISPR–Cas9 to double‐edit the C‐C chemokine receptor type V (CCR5) and HIV‐1 viral DNA genes after administration of the drug therapy, and the combination of CRISPR and drug therapy produced highly efficient elimination of HIV‐1 proviral DNA and restoration of the relative number of human CD4 T cells without detectable residual HIV‐1 DNA fragments, which demonstrates the potential of gene editing technology in combination with drugs to eliminate HIV 1 infection in vivo.^381^ The smaller endonuclease Cas12b has the advantage of high sequence specificity generates larger deletions and could be used for targeted genome editing in mammalian cells. It has been demonstrated that spCas9 and Cas12a could attack the integrated viral DNA genome in cells and effectively inhibit HIV infection.^382^ The study further explored the inhibition of cell‐transmitted HIV infection by CRISPR–Cas12b using anti‐HIV gRNAs, and the results showed that Cas12b could achieve complete inactivation of HIV with only one gRNA and that the use of two gRNAs could result in a significant increase in the anti‐HIV efficacy of Cas12b, providing a scientific rationale for CRISPR–Cas12b‐mediated HIV‐1 inactivation.^383^ Another study confirmed that CRISPR–Cas13a could target cut the HIV RNA genome and mRNA, reduce the level of newly synthesized viral RNA, and destroy viral RNA that enters the cellular viral capsid, thus inhibiting cellular infection with HIV‐1, and is a promising gene editing tool for HIV therapy.^384^

## 5 US FDA‐APPROVED CLINICAL TRIALS OF GENE THERAPY FOR HUMAN DISEASES

Gene editing technology based on CRISPR–Cas9 is already being used in clinical trials on human diseases, and the US FDA has now approved gene therapy trials for several human diseases (Table 4). The first patient with SCD to receive an experimental treatment using autologous HSPC gene editing to correct their sickle cell mutation and then transfusing the HSPCs back into the body now produces few sickle RBCs and no pathological manifestations such as severe pain, providing confidence in the clinical application of gene therapy (Figure 4C).^385^ At present, some clinical trials are testing gene‐editing methods. CTX001, a gene‐editing treatment for SCD and transfusion‐dependent thalassemia, has been approved by the US FDA and is currently in phase 1/2/3 trials (NCT03655678, NCT03745287, NCT05477563, NCT05329649, and NCT04208529). CTX001 reduces *BCL11A* expression in erythrocytes, restores γ‐globin synthesis, and produces high levels of HbF in erythrocytes by gene editing patients’ HSCs in vitro with CRISPR–Cas9, thereby re‐infusing the gene‐edited HSCs into patients to stimulate the synthesis of HbF in the patients, to restore the normal functioning of their RBCs for the treatment of severe SCD and β‐thalassemia. CTX001 has been renamed exagamglogene autotemcel therapy (Exa‐Cel) and received orphan drug designation in the United States as a one‐time therapy.^386^ Exa‐Cel (NCT05951205) has eliminated severe vascular occlusion during the interim treatment of patients with SCD and can potentially become the first CRISPR gene‐editing therapy to receive regulatory approval worldwide.^387^ After changing its name to Exa‐Cel and then again to Casgevy over the past few months, CTX001 is intended for the treatment of SCD patients 12 years of age and older with recurrent vaso‐occlusive crises (VOCs), as well as patients with TDT for whom matched human leukocyte antigens (HLAs) are unavailable and who are unsuitable for HSCT therapy. Results from clinical trials have shown Casgevy has the potential to provide a functional cure, and patients are still undergoing long‐term evaluation of the safety and efficacy of Casgevy.^388^ In November 2023, the UK Medicines and Healthcare products Regulatory Agency was the first to approve Casgevy for the treatment of SCD and TDT, making Casgevy the first CRISPR gene‐edited drug approved for marketing in the world.^389^ This was a landmark approval for CRISPR therapies. In December of the same year, the US FDA approved the marketing of Casgevy for the treatment of SCD,^390^ and the European Medicines Agency (EMA) approved Casgevy for the treatment of SCD and TDT. The approval of Casgevy demonstrates the potential for the expansion of gene therapy applications in human disease and offers hope for the development of next‐generation gene editing technologies. In two global clinical trials of Casgevy for the treatment of patients with SCD and TDT, the trials met their respective primary endpoints of patients being free of severe VOC or not requiring blood transfusions for at least 12 consecutive months.^215^ Previously announced clinical trial results demonstrated Casgevy's potential to provide a functional cure with a single treatment. A total of 42 ot of the 44 TDT patients treated with Casgevy no longer required transfusions at a follow‐up time of 1.2–37.2 months, and the remaining two patients received transfusions at levels that were 75 and 89% lower, respectively. All 31 treated patients with severe SCD had no VOC at a follow‐up time of 2.0–32.3 months. The first patient with SCD to receive Casgevy therapy remains HbF in approximately 46% of her blood and carries this protein in 99.7% of her RBCs nearly a year after treatment. In addition, a biopsy of the patient's bone marrow cells showed that more than 81% of the cells contained the genetic changes needed to produce HbF, indicating that the genetically edited cells were alive and functioning in her body.^385^ Since this patient's transplant, she no longer requires blood transfusions and her pain has disappeared. There have been no reported negative side effects in patients treated with Casgevy. As an approved gene editing therapy, Casgevy utilizes CRISPR–Cas9‐based gene editing technology to enable the successful clinical application of gene therapy. The excellent outcome and subsequent prognosis of the patients confirm the safety and feasibility of Casgevy as a curative gene therapy option for SCD while providing a scientific reference for CRISPR–Cas9 editing of patients' HSCs.

**TABLE 4:** Clinical trial of CRISPR‐based gene editing technology for human diseases.

Diseases	Study title	Intervention/treatment	CRISPR/Cas system	Study phase	Therapeutic principle	Clinical treatment effect	First posted time	Recruitment status	References/ClinicalTrials.gov identifier
SCD	Evaluation of safety and efficacy of CTX001 in pediatric participants with severe sickle cell disease (SCD)	CTX001 (exagamglogene autotemcel Exa‐cel)	CRISPR–Cas9	Phase 3	Autologous CD34+ hHSPCs modified with CRISPR–Cas9 at the erythroid lineage‐specific enhancer of the BCL11A gene	Clinical trials still in progress	April 15, 2022	Recruiting	NCT05329649
	Evaluation of safety and efficacy of CTX001 in adolescent and adult participants with severe SCD, βS/βC genotype (HbSC)	CTX001 (exagamglogene autotemcel Exa‐cel)	CRISPR–Cas9	Phase 3	Autologous CD34+ hHSPCs modified with CRISPR–Cas9 at the erythroid lineage‐specific enhancer of the BCL11A gene	Clinical trials still in progress	July 18, 2023	Not yet recruiting	NCT05951205
	A safety and efficacy study evaluating CTX001 in subjects with severe SCD	CTX001 (exagamglogene autotemcel Exa‐cel)	CRISPR–Cas9	Phase 2 Phase 3	Autologous CD34+ hHSPCs modified with CRISPR–Cas9 at the erythroid lineage‐specific enhancer of the BCL11A gene	After transplantation of CRISPR–Cas9‐edited autologous CD34+ cells targeting the BCL11A enhancer, SCD patients with βS/βS and a single α‐bead protein deletion had high levels of allelic editing in both bone marrow and blood, and the patients’ fetal hemoglobin levels increased to 43.2% and sickle hemoglobin to 52.3% at month 15.	November 19, 2018	Active, not recruiting	NCT03745287^215^, ^405^, ^406^
	Study of safety and efficacy of genome‐edited hematopoietic stem and progenitor cells in sickle cell disease (SCD)	OTQ923	CRISPR–Cas9	Phase 1 Phase 2	OTQ923 is a genome‐edited autologous hematopoietic stem and progenitor cell (HSPC) product that increases fetal hemoglobin (HbF) and reduces complications of sickle cell disease by decreasing the biological activity of BCL11A.	Clinical trials still in progress	June 23, 2020	Recruiting	NCT04443907
	Transplantation of clustered regularly interspaced short palindromic repeats modified hematopoietic progenitor stem cells (CRISPR_SCD001) in patients with severe SCD	CRISPR_SCD001	CRISPR–Cas9	Phase 1 Phase 2	Single infusion of CRISPR/Cas9‐edited sickle allele‐modified differentiated cluster (CD34+) hematopoietic stem progenitor cells (HSPC) in subjects with severe sickle cell disease (SCD) aged ≥12–35 years.	Clinical trials still in progress	March 1, 2021	Not yet recruiting	NCT04774536
	Gene correction in autologous CD34+ hematopoietic stem cells (HbS to HbA) to treat severe SCD (CEDAR)	GPH101 drug product	CRISPR–Cas9	Phase 1 Phase 2	GPH101 drug product is a human autologous CRISPR–Cas9 edited and sickle mutation‐corrected HSPC product.	Clinical trials still in progress	March 29, 2021	Terminated	NCT04819841
	A study evaluating the safety and efficacy of EDIT‐301 in participants with severe SCD (RUBY)	EDIT‐301	CRISPR/Cas12a	Phase 1 Phase 2	EDIT‐301 utilizes autologous CRISPR gene‐edited CD34+ hematopoietic stem cells administered via a single intravenous infusion for the treatment of SCD.	Clinical trials still in progress	April 21, 2021	Recruiting	NCT04853576
	BEACON: A study evaluating the safety and efficacy of BEAM‐101 in patients with severe SCD (BEACON)	BEAM‐101	CRISPR–Cas9 (adenine base editor)	Phase 1 Phase 2	BEAM‐101 was manufactured with plerixafor mobilized collected autologous CD34+ hematopoietic stem cells with CRISPR–Cas9‐based gene editing in vitro and administered as a single dose by intravenous infusion.	Clinical trials still in progress	July 13, 2022	Recruiting	NCT05456880
β‐Thalassemia and SCD	A long‐term follow‐up study of subjects with β‐thalassemia or SCD treated with autologous CRISPR–Cas9 modified hematopoietic stem cells (CTX001)	CTX001 (exagamglogene autotemcel Exa‐cel)	CRISPR–Cas9	Phase 3	All participants who complete or discontinue one of the multiple parent studies after CTX001 infusion will be asked to participate in this long‐term follow‐up study.	Clinical trials still in progress	December 23, 2019	Enrolling by invitation	NCT04208529
	Evaluation of efficacy and safety of a single dose of CTX001 in participants with transfusion‐dependent β‐thalassemia and SCD	CTX001 (exagamglogene autotemcel Exa‐cel)	CRISPR–Cas9	Phase 3	Autologous CD34+ hHSPCs modified with CRISPR–Cas9 at the erythroid lineage‐specific enhancer of the BCL11A gene	Clinical trials still in progress	July 28, 2022	Recruiting	NCT05477563
β‐Thalassemia	A single‐arm, open‐label, multisite, single‐dose Phase 1/2/3 study in subjects with transfusion‐dependent β‐thalassemia (TDT)	CTX001 (exagamglogene autotemcel Exa‐cel)	CRISPR–Cas9	Phase 2 Phase 3	Autologous CD34+ hHSPCs modified with CRISPR–Cas9 at the erythroid lineage‐specific enhancer of the BCL11A gene	After transplantation of CRISPR–Cas9 edited autologous CD34+ cells targeting the BCL11A enhancer, β‐thalassemia patients with the β0/β (IVS‐I‐110) genotype had high levels of allele editing in both bone marrow and blood, and hemoglobin levels returned to normal by month 4.	August 31, 2018	Active, not recruiting	NCT03655678^215^, ^405^
	A single‐dose, open‐label study in pediatric participants with TDT.	CTX001 (exagamglogene autotemcel Exa‐cel)	CRISPR–Cas9	Phase 3	Autologous CD34+ hHSPCs modified with CRISPR–Cas9 at the erythroid lineage‐specific enhancer of the BCL11A gene	Clinical trials still in progress	May 2, 2022	Recruiting	NCT05356195
	EDIT‐301 for autologous hematopoietic stem cell transplant (HSCT) in participants with transfusion‐dependent beta thalassemia (TDT)	EDIT‐301	CRISPR/Cas12a	Phase 1 Phase 2	EDIT‐301 utilizes autologous CRISPR gene‐edited CD34+ hematopoietic stem cells administered via a single intravenous infusion for the treatment of TDT.	Clinical trials still in progress	July 6, 2022	Recruiting	NCT05444894
	Phase 1/2a, single dose study investigating NTLA‐5001 in subjects with acute myeloid leukemia (AML)	NTLA‐5001	CRISPR–Cas9	Phase 1 Phase 2	Autologous WT1‐directed TCR T cells engineered ex vivo using CRISPR/Cas9 as intravenous infusion after preconditioning chemotherapy.	Transgene in all collected patients’ samples were below limit of quantification or negative	October 4, 2021	Terminated (Pivoting to an allogeneic version of this program currently in preclinical development.)	NCT05066165
AML	CRISPR‐edited allogeneic anti‐CLL‐1 CAR‐T cell therapy in patients with relapsed/refractory acute myeloid leukemia (AMpLify)	CB‐012	CRISPR–Cas12a chRDNA	Phase 1	CB‐012 allogeneic CAR‐T cell therapy targeting CLL‐1 cyclophosphamide and fludarabine chemotherapy for lymphodepletion	Clinical trials still in progress	November 13, 2023	Recruiting	NCT06128044
	A long‐term follow‐up study of patients who received VOR33	VOR33	CRISPR–Cas9	–	VOR33 is an allogeneic CRISPR/Cas9 genome‐edited hematopoietic stem and progenitor cell therapy product that lacks the CD33 myeloid protein.	Clinical trials still in progress	April 4, 2022	Recruiting	NCT05309733
	llogeneic engineered hematopoietic stem cell transplant (HCT) lacking the CD33 protein, and post‐HCT treatment with mylotarg, for patients with CD33+ AML	Biological: VOR33 Drug: Mylotarg	CRISPR–Cas9	Phase 1 Phase 2	Participants will undergo a myeloablative HCT with matched related or unrelated donor CD34+‐selected hematopoietic stem and progenitor cells (HSPCs) engineered to remove CD33 expression (VOR33 product). Mylotarg™ will be given after engraftment for up to four cycles.	Clinical trials still in progress	April 19, 2021	Recruiting	NCT04849910^407^
Multiple myeloma	CRISPR‐edited allogeneic anti‐BCMA CAR‐T cell therapy in patients with relapsed/refractory multiple myeloma (CaMMouflage)	Biological: CB‐011	CRISPR–Cas12a chRDNA	Phase 1	Escalation with CB‐011 in ascending doses using a traditional 3+3 design	Clinical trials still in progress	February 10, 2023	Recruiting	NCT05722418^396^
	A safety and efficacy study evaluating CTX120 in subjects with relapsed or refractory multiple myeloma	Biological: CTX120	CRISPR–Cas9	Phase 1	Administered by IV infusion following lymphodepleting chemotherapy	Clinical trials still in progress	January 28, 2020	Active, not recruiting	NCT04244656^394^
Lymphoma	CRISPR‐edited allogeneic anti‐CD19 CAR‐T cell therapy for relapsed/refractory B cell non‐Hodgkin lymphoma (ANTLER)	Genetic: CB‐010 Drug: Cyclophosphamide Drug: Fludarabine	Cas9–chRDNA	Phase 1	Patients with relapsed or refractory non‐Hodgkin lymphoma will receive CB‐010 following lymphodepletion.	Clinical trials still in progress	November 20, 2020	Recruiting	NCT04637763^398^
	A safety and efficacy study evaluating CTX110 in subjects with relapsed or refractory B‐cell malignancies (CARBON)	Biological: CTX110	CRISPR–Cas9	Phase 1 Phase 2	Administered by IV infusion following lymphodepleting chemotherapy	Clinical trials still in progress	July 29, 2019	Active, not recruiting	NCT04035434
	A safety and efficacy study evaluating CTX112 in subjects with relapsed or refractory B‐cell malignancies	Biological: CTX112	CRISPR–Cas9	Phase 1 Phase 2	Administered by IV infusion following lymphodepleting chemotherapy	Clinical trials still in progress	December 9, 2022	Recruiting	NCT05643742
	A safety and efficacy study evaluating CTX130 in subjects with relapsed or refractory T or B cell malignancies (COBALT‐LYM)	Biological: CTX130	CRISPR–Cas9	Phase 1	Patients receive 3 days of lymphocyte‐clearing chemotherapy consisting of fludarabine at 30 mg/m^2^/day and cyclophosphamide at 500 mg/m^2^/day followed by a single infusion of CTX130.	Clinical trials still in progress	August 6, 2020	Active, not recruiting	NCT04502446
Solid tumors	A phase I/II trial in patients with metastatic gastrointestinal epithelial cancer administering tumor‐infiltrating lymphocytes in which the gene encoding CISH was inactivated using the CRISPR/Cas9 system	Drug: Cyclophosphamide Drug: Fludarabine Biological: Tumor‐infiltrating lymphocytes (TIL) Drug: Aldesleukin	CRISPR–Cas9	Phase 1 Phase 2	Phase I: nonmyeloablative, lymphodepleting preparative regimen of cyclophosphamide and fludarabine +escalating doses of CISH inactivated TIL + high‐dose aldesleukin	Clinical trials still in progress	June 11, 2020	Recruiting	NCT04426669^400^, ^401^
	CISH inactivated TILs in the treatment of non‐small cell lung cancer (NSCLC) (CheckCell‐2)	Drug: Fludarabine Drug: Cyclophosphamide Biological: CISH inactivated TIL Drug: Aldesleukin Drug: Pembrolizumab	CRISPR–Cas9	Phase 1 Phase 2	Nonmyeloablative, lymphodepleting preparative regimen of cyclophosphamide and fludarabine +escalating doses of CISH inactivated TIL + high‐dose aldesleukin	Clinical trials still in progress	October 4, 2022	Active, not recruiting	NCT05566223
	A safety and efficacy study evaluating CTX131 in adult subjects with relapsed or refractory solid tumors	Biological: CTX131	CRISPR–Cas9	Phase 1 Phase 2	Administered by IV infusion following lymphodepleting chemotherapy	Clinical trials still in progress	April 3, 2023	Recruiting	NCT05795595
	A safety and efficacy study evaluating CTX130 in subjects with relapsed or refractory renal cell carcinoma (COBALT‐RCC)	Biological: CTX130	CRISPR–Cas9	Phase 1	Patients received 3 days of lymphodepleting chemotherapy, consisting of fludarabine at 30 mg/m^2^/day and cyclophosphamide at 500 mg/m^2^/day, followed by a single CTX130 infusion.	Clinical trials still in progress	June 18, 2020	Active, not recruiting	NCT04438083^402^
LCA10	Single ascending dose study in participants with leber congenital amaurosis 10 (LCA10)	Drug: EDIT‐101	CRISPR–Cas9	Phase 1 Phase 2	Participants will receive a single dose of EDIT‐101 administered via subretinal injection in the study eye.	Clinical trials still in progress	March 13, 2019	Active, not recruiting	NCT03872479
DMD	Treatment of a single patient with CRD‐TMH‐001	Drug: CRD‐TMH‐001	CRISPR–Cas9	Phase 1	Participant will receive a single dose of CRD‐TMH‐001 administered via intravenous injection.	This 27‐year‐old man with advanced DMD had severe cardiopulmonary toxic effects within 6 days after intravenous administration of rAAV9–dSaCas9–VP64 at a dose of 1 × 10^14^ vg per kg. In the end, the patient unfortunately died.	August 24, 2022	Active, not recruiting	NCT05514249
Diabetes mellitus	An open‐label, FIH study evaluating the safety and tolerability of VCTX210A combination product in subjects with T1D	Combination Product: VCTX210A unit	CRISPR–Cas9	Phase 1	CRISPR–Cas9 genetically modified PEC210A cells loaded into a delivery device, up to seven (7) units will be implanted.	Clinical trial results not yet uploaded	January 27, 2022	Completed	NCT05210530
	An open‐label, FIH study evaluating the safety, tolerability, and efficacy of VCTX211 combination product in subjects With T1D	Combination product: VCTX211	CRISPR–Cas9	Phase 1 Phase 2	CRISPR–Cas9 genetically modified PEC211 cells loaded into a delivery device	Clinical trials still in progress	October 4, 2022	Recruiting	NCT05565248
HIV‐1	Study of EBT‐101 in Aviremic HIV‐1 infected adults on stable ART	Biological: EBT‐101	CRISPR–Cas9	Phase 1	On day 1, eligible participants will receive a single IV dose of EBT‐101. All participants will be assessed for eligibility for an analytical treatment interruption (ATI) of their background ART at week 12. All participants will be followed through week 48 (end of study).	Clinical trials still in progress	December 3, 2021	Recruiting	NCT05144386
	Long‐term follow‐up study of HIV‐1 infected adults who received EBT‐101	Biological: EBT‐101	CRISPR–Cas9	Phase 1	Long‐term follow up of participants who received EBT‐101	Clinical trials still in progress	December 3, 2021	Enrolling by invitation	NCT05143307

*Data sources*: clinical registration website “https://clinicaltrials.gov/.”

A Phase I/II clinical study is evaluating the security and efficacy of the CRISPR–Cas9‐based HSPC editing product OTQ923 in patients with SCD (NCT04443907). OTQ923 was administered to adults and children with SCD aged 2–17 years. A single intravenous infusion of the OTQ923 cell suspension was expected to decrease BCL11A biological activity, increase HbF, and reduce SCD complications. CRISPR_SCD001 has been approved by the US FDA for clinical trials (NCT04774536). CRISPR_SCD001 uses CRISPR–Cas9 to correct the sickle allele in CD34^+^ HSPCs and is given as a single intravenous HSCT infusion to patients with SCD aged 12–35 years. CEDAR is a clinical trial to treat severe SCD by genetically modifying HSCs (NCT04819841). The GPH101 drug given in the CEDAR trial is an HSPC product with human autologous CRISPR–Cas9 editing and sickle mutation correction and has been assigned orphan drug status by the US FDA. GPH101 permanently minimized HbS production by correcting inherited β‐globin gene mutations and restoring HbA expression. EDIT‐301 is a drug product for the autologous gene editing of HSCs. It uses CRISPR–AsCas12a to edit the *HBG1* and *HBG2* promoters for autologous HSPC transplantation into patients with severe SCD and β‐thalassemia (NCT04853576). EDIT‐301 is designed to disrupt BCL11A binding sites, improve disease symptoms, increase HbF expression, and treat SCD and β‐thalassemia by mimicking the natural HPFH mechanisms. BEAM‐101 is a single‐base editing therapy used to treat SCD. It involves the autologous base editing of CD34^+^ HSPCs in vitro using a CRISPR–Cas9‐based ABE (NCT05456880). The ABE combines A‐to‐G base editing in the *HBG1* and *HBG2* gene promoters that regulate HbF expression, mimicking naturally occurring HbF‐inducing mutations and reactivating HbF expression through high‐level base editing to compensate for the lack of HbA in patients with SCD. These ongoing clinical trials show that CRISPR–Cas9‐based gene‐editing techniques can reactivate HbF expression and have the potential to be soon used in the clinical treatment of patients with SCD and bring hope for the long‐term cure of SCD.^391^

In immunotherapy for AML, the lack of tumor‐specific antigens can lead to targeted nontumor toxicity, the study used CRISPR–Cas9 to knockdown CD33 from healthy donor HSPC to generate the HSPC transplant product tremtelectogene empogeditemcel (trem‐cel, formerly known as VOR33, NCT04849910). Trem‐cel, an allogeneic gene‐edited product matched to HLAs, could treat AML patients at high risk of leukemic relapse and posttransplant mortality, with the promise of mitigating the hematologic toxicity associated with anti‐CD33 cell therapy. Mouse models have confirmed the long‐term implantation, multispectral differentiation, and durability of trem‐cel, and the US FDA has approved a clinical trial utilizing trem‐cel to evaluate its safety and efficacy in AML patients.^392^ CB‐012 (NCT06128044) is a gene editing technology from Caribou Biosciences using in‐house Cas12a chRDNA gene editing technology, CLL‐1 (CD371) is expressed on the surface of AML tumor cells and leukemia stem cells, but not on normal HSCs and could be a therapeutic target for AML. The study constructed allogeneic anti‐CLL‐1 CAR‐T cell therapy for the treatment of relapsed or refractory AML (r/r AML) by five edits: TRAC KO, human anti‐CLL‐1 CAR‐specific insertion into the TRAC gene, PD‐1 KO, B2M‐HLA‐E insertion, and endogenous B2M KO. CB‐012, which uses checkpoint disruption and immune camouflage to avoid being attacked by the immune system in the patient's body and to enhance antitumor capabilities, has been approved by the US FDA for Phase I clinical trials. The nephroblastoma 1 (WT1) antigen is overexpressed in more than 90% of AML, Intellia's investigational therapy, NTLA‐5001 (NCT05066165), is a T‐cell therapy targeting WT1 for the treatment of AML. NTLA‐5001 uses gene editing technology to remove the innate TCR of T cells while introducing a high‐affinity TCR targeting WT1 with potent anticancer activity. NTLA‐5001 has been approved for clinical evaluation and has been granted Orphan Drug Status designation by the US FDA in 2022, making it a future therapeutic option for AML patients.

CAR T‐cell therapies have antitumor activity in lymphoma, leukemia, and MM.^393^ CTX120 (NCT04244656) is an allogeneic CAR T therapy developed by CRISPR Therapeutics, Inc. that targets and edits the B cell maturation antigen (BCMA) of a healthy donor via CRISPR–Cas9, CTX120 has been used in clinical trials to evaluate its efficacy and safety in the treatment of MM.^394^ CRISPR Therapeutics has also developed an allogeneic CAR T cell therapy for MM, CaMMouflage (CB‐011, NCT05722418).^395^ CB‐011 is a low‐immunogenic, allogeneic anti‐BCMA CAR T‐cell therapy candidate that utilizes Cas12a CRISPR hybridization of RNA‐DNA (chRDNA) and gene knock‐in to achieve four genomic alterations in CB‐011 to healthy donor‐sourced T‐cells, CB‐011 could eliminate allogeneic allogeneic reactivity, reduced susceptibility to NK cell elimination, and cells exhibited inhibitory recognition and cytotoxicity against HLA mismatched T cells, CB‐011 has received US FDA orphan drug designation and clinical trials are underway to explore its therapeutic efficacy in MM.^396^

CRISPR hybridized RNA‐DNA (chRDNA) could improve Cas9 specificity while retaining target editing activity.^397^ In response to checkpoint interference, the study utilized Cas9 chRDNA knockdown of TRAC to remove the TCR, insertion of CD19 CAR specificity into the TRAC gene, and KO of PDCD1, which encodes the PD‐1 checkpoint receptor, to develop CB‐010, a therapeutic for homozygous anti‐CD19 CAR‐T cells (NCT04637763). CB‐010 for the treatment of relapsed or refractory B‐cell non‐Hodgkin's lymphoma (r/r B‐NHL) has been granted orphan drug designation by the US FDA and is currently in clinical trials.^398^ CTX110 (NCT04035434) is a targeted CD19 CAR‐T cell therapy constructed by CRISPR Therapeutics utilizing CRISPR/Cas9 to study the precise insertion of CAR structures into the TRAC locus to improve safety, knockdown of the TCR utilizing CRISPR/Cas9 to reduce the risk of GvHD, and elimination of the MHC class I expressed on the surface of CAR‐T cells to alleviate the immune rejection of CTX110 by T cells from patients with LBCL. Phase I results from CRISPR Therapeutics' CARBON trial showed that higher‐dose CTX110 single‐agent treatment achieved a 58% overall remission rate and a 38% complete remission rate, making it a potential treatment option for patients with LBCL. CTX110 is a first‐generation B‐cell lymphoma therapeutic candidate targeting CD19, and CTX112 (NCT05643742) builds on the foundation of CTX110 by editing the genes encoding Regnase‐1 and TGFBR2 using CRISPR/Cas9 to increase functional persistence while avoiding immunosuppression of CAR T‐cell activity. In a xenograft model, CTX112 is 10 times more effective than CTX110 and is an emerging therapeutic option for B‐cell lymphoma treatment. For relapsed or refractory RCC and various lymphoma subtypes, CTX130 (NCT04502446) is an effective therapeutic strategy developed by CRISPR Therapeutics, Inc.CTX130 is a gene‐edited allogeneic CAR‐T cell therapy from a healthy donor targeting CD70, which is expressed in a wide range of solid and hematologic tumors The Phase 1 COBALT‐LYM trial is focused on evaluating the safety and efficacy of CTX130 for the treatment of patients with relapsed or refractory T‐cell lymphoma. CTX130 for the treatment of T‐cell lymphoma has received orphan drug designation from the US FDA.

Immunotherapies based on gene editing techniques mainly modify T cells to recognize tumor antigens and increase antitumor activity.^399^ It was found that TILs from patients with metastatic GI tract contained CD4(+) and/or CD8(+) T cells.^400^ Cytokine‐induced SH2 protein (CISH), a member of the cytokine signaling inhibitor (SOCS) family CISH is a novel intracellular immune checkpoint that regulates T‐cell signaling and function, and studies have shown that knockdown of CISH in CD8(+) T cells using CRISPR/Cas9 improves the immunotherapeutic efficacy of cancer.^401^ A study genetically engineered antigen‐specific TIL to inhibit the expression of the checkpoint CISH in T cells by efficiently editing the genes encoding the intracellular checkpoint target CISH using CRISPR/Cas9 (NCT04426669), inhibiting the antitumor activity of lymphocytes from patients with metastatic cancers, in order to evaluate the genetically engineered T cell therapy safety and efficacy in treating patients with solid tumors of the GI tract in the context of novel checkpoint inhibition. CheckCell‐2 (NCT05566223), a therapeutic approach based on the CRISPR/Cas9‐encoded CISH gene, was also applied in a clinical trial in patients with metastatic NSCLC.CD70, which has limited expression in normal tissues and is expressed in a wide range of solid tumors and hematological tumors, is able to efficiently modulate T cells and promote immunosuppression in the tumor microenvironment, and it could be used as a target for immunotherapy.^402^ CTX131 (NCT05795595) is a targeted allogeneic CD70 CAR T‐cell immunotherapy developed by CRISPR Therapeutics, Inc. whereby CRISPR/Cas9 modifies T‐cells from a healthy donor in vitro to enhance the therapeutic efficacy of CAR T in relapsed and refractory solid tumors. CTX131 is being used in clinical trials to evaluate the safety and efficacy of its clinical application. CTX130 (NCT04438083), another CD70 CAR T cell immunotherapy developed by CRISPR Therapeutics, should be used in patients with advanced, relapsed, or refractory RCC.

Allergan and Editas Medicine have teamed up to develop the gene‐editing drug EDIT‐101 (NCT03872479) for the treatment of patients with LCA10. EDIT‐101 utilizes CRISPR/Cas9 to remove the aberrant splice donor created by the IVS26 mutation in the CEP290 gene, restoring CEP290 expression to normal. EDIT‐101 demonstrated sustained and rapid gene editing in mice, and therapeutic gene editing was also achieved in comparable alternative NHP vectors.^403^, ^404^ EDIT‐101 has been approved for use in clinical trials and holds promise for eliminating the effects of disease‐causing mutations in patients, improving photoreceptor cell function, and enabling safe and effective therapies.

Cure Rare Disease has developed CRD‐TMH‐001 (NCT05514249), which focuses on stabilizing or reversing DMD by delivering the CRISPR gene editing system via AAV to act on the muscle promoter and exon 1 mutations on the protein gene to help patients make the muscle protein that the body lacks (full‐length antimyasthenic proteins) DMD symptom progression. CRD‐TMH‐001 is the first human trial of a CRISPR therapy designed to use gene editing technology to change the way the body responds to the existing genetic code and is the first trial of a personalized CRISPR therapy and the first trial of a gene editing technology for DMD. CRD‐TMH‐001 was the first DMD gene‐editing therapy to receive US FDA approval for clinical trials. CRD‐TMH‐001 delivers loss‐of‐cleavage activity Cas9 and transcriptional activator protein VP64 from Staphylococcus aureus with rAAV9 to up‐regulate nonmuscle full‐length antimorphic protein isoform (Dp427c) expression. The only 27‐year‐old patient with advanced DMD, Terry Horgan, received a daily intravenous dose of rAAV9–dSaCas9–VP64 at a dose of 1 × 10^14^ vg per kg. One day after vector delivery, the patient developed ventricular premature beats, hypercapnia with respiratory acidosis occurred on days 3–4, cardiac function deteriorated on day 5, acute respiratory distress and left ventricular systolic dysfunction developed on day 6, and the patient died of multiorgan failure 8 days later. Disappointingly, the AAV vector triggered a strong immune response, resulting in the loss of the patient's opportunity to benefit from gene therapy. CRISPR–Cas system‐mediated gene editing has inherently difficult to control off‐target editing, and there may be safety issues with the AAV vector delivery system, as demonstrated by the patient's death, and the fact that in patients with advanced DMD, high doses of rAAV can elicit a strong innate immune response leading to acute respiratory distress syndrome. The patient's death triggered a rethinking and a focus on the safety of gene editing therapies, a more cautious application of AAV vectors in subsequent clinical trials, and an improvement in the delivery technology of the gene editing system and the optimization of the gene editor itself, to prevent lethal immunotoxic effects and avoid a recurrence of the tragedy.^174^, ^175^

T1D is an autoimmune disease that begins when a human's own immune cells attack and destroy insulin‐producing beta cells in the pancreas, resulting in a lack of beta cells, a cessation of insulin production, and uncontrolled blood sugar levels. The current standard treatment for T1D is insulin replacement therapy and regular insulin injections, and no curative treatment has been developed. Two companies, CRISPR Therapeutics and ViaCyte, have codeveloped the CRISPR/Cas9 gene‐edited allogeneic VCTX210 (NCT05210530) based stem cell therapy for gene therapy of T1D and insulin‐dependent T2D. VCTX210, the first gene therapy for T1D, consists of CRISPR/Cas9‐modified allogeneic pancreatic endodermal cells (PEC210A) capable of promoting immune evasion and survival with a PEC‐Direct pouch that delivers and retains the PEC210A cells. The PEC‐Direct pouch allows vascular access and interaction with the implanted cells, which ensures the stability of the implanted cells and allows the released insulin to enter the circulation smoothly. VCTX210 has a long‐lasting immune escape, eliminating the need for immunosuppressive treatments and allowing the patient to regain insulin secretion and achieve a functional cure. VCTX211 (NCT05565248) is a second‐generation T1D gene therapy strategy developed by CRISPR Therapeutics and ViaCyte, and both VCTX210 and VCTX211 have been approved by the US FDA for clinical trials evaluating the safety and efficacy of transplanting stem cell‐derived cells into patients.

EBT‐101 (NCT05144386, NCT05143307) is a CRISPR–Cas9‐based double‐cut gene therapy designed to cure HIV infection with a single intravenous infusion. EBT‐101 utilizes AAV delivery of CRISPR–Cas9 and two gRNAs to achieve multiple in vivo editing by making two cuts in the integrated retroviral DNA to minimize potential viral escape and replication, to completely disrupt the HIV viral genome, which results in the complete eradication of the HIV. EBT‐101 is the first clinical trial of multiple in vivo gene editing applied to infectious diseases, administered intravenously to viremia‐free HIV‐1‐infected adults on stable ART to assess its safety and tolerability in adult HIV‐1 patients.

## 6 THE CHALLENGES OF APPLYING GENE EDITING TECHNOLOGY TO CLINICAL TREATMENTS

The CRISPR–Cas system is a gene‐modifying tool to manipulate the genome for study and therapy. Gene editing technology has changed gene therapy from delivering exogenous genes to the precise gene editing of the human genome, with the potential to be used in preclinical research and clinical treatment of human diseases.^154^, ^215^ Despite advances in CRISPR–Cas‐based genome engineering techniques, there remain some challenges in translating these tools into the clinic, the potential limitations and safety concerns of gene editing technologies must be overcome.^215^ As the CRISPR–Cas system‐based gene editing technology enters the clinic, many challenges, such as off‐target events and the safety of in vivo delivery modes, have hindered gene editing development and utilization in clinical treatment. The safety of gene editing is a crucial test of the CRISPR–Cas system for clinical application.^408^ To better apply gene editing technology to clinical treatment, in‐depth research has been conducted on how to detect the CRISPR–Cas system's off‐target effect, select appropriate in vivo gene delivery methods, and reduce the off‐target rate by improving the gene editing system and optimizing in vivo gene delivery methods; to use gene editing technology to achieve the best efficacy and minimum safety problems; and to realize the secure and effective clinical application of gene editing technology in human disease treatment.^408^

### 6.1 Off‐target

CRISPR–Cas9‐based gene editing is a powerful driver of advances in life sciences and medicine because of the promise of permanent genetic modification with a single injection of CRISPR–Cas9.^241^ The safety of genome editing is seriously compromised by off‐target mutagenesis.^409^ Off‐target editing in other genomic locations that are highly homologous to gRNA targeting sequences may result in unexpected gene mutation, gene insertion, gene deletion, and other problems, resulting in abnormal chromosome structure products and damage to the function of normal genes.^410^ Such changes can potentially cause genotoxicity and unpredictable harm during human disease therapy, hindering the CRISPR–Cas system's clinical translation process.^411^ Therefore, prediction and quantification are essential for designing experimental genome editing and evaluating the safety of therapeutic genome editing programs.^412^ Developing gene editing strategies that specifically recognize gene mutations in patients is promising to increase the targeting of edited mutant alleles, reduce the incidence of off‐target events, and improve the safety of gene editing.

#### 6.1.1 Three types of commonly used off‐target prediction tools

Three primary techniques exist for genome‐wide detection of potential off‐target sites induced by nucleases: sequence‐homology‐based bioinformatics prediction, cell‐capture‐based off‐target editing events, and using genomic DNA templates for cleavage site characterization in vitro.^413^ These approaches are used for predicting or identifying off‐target effects in gene editing, detecting gene editing outcomes, and assessing the safety of gene editing (Table 5).^411^ The commonly used computer prediction techniques mainly include E‐CRISP,^414^ COSMID,^415^ Cas‐OFFinder,^416^ CRISPRseek,^417^ CCTOP (Consensus Constrained TOPology prediction),^418^ CROP‐IT (a web‐based CRISPR/Cas9 Off‐target Prediction and Identification Tool),^419^ CHOPCHOP,^420^ CHOPCHOP v2,^421^ CHOPCHOP v3,^422^ Crisflash,^423^ CRISPOR,^424^ and CRISTA.^425^ Computational prediction algorithms are powerful and convenient but often miss most confirmed off‐target sites. They are also ineffective in ranking active off‐target sites within cells.^413^

**TABLE 5:** Methods for assessing genome‐wide specificity of the CRISPR–Cas system.

	Method name	Strengths	Limitations	Website	References
Bioinformatics‐based tools	E‐CRISP	For gRNA sequence design, also for off‐target effects and target site homology assessment	The accuracy of off‐target site detection may be hampered by the effects of genomic environment and DNA modifications.	http://www.e‐crisp.org/E‐CRISP/	414
	COSMID	Potential off‐target sites can be searched in the genome for the identification and quantification of CRISPR–Cas‐induced cellular off‐target cleavage, and a ranked list of potential off‐target sites facilitates the selection and evaluation of prospective target sites.	Due to limited experimental results, it is not possible to quantitatively consider the effect of insertion or deletion sites on the off‐target rate, including their number, location and combination.	https://crispr.bme.gatech.edu/	222
	CasOT	A genome‐wide Cas9 gRNA off‐target search tool that can find potential off‐target sites in any given genome or user‐supplied sequence, with several adjustable parameters such as PAM type and number of mismatches in seeded and nonseeded regions.	Longer time consuming, no bulges allowed.	http://eendb.zfgenetics.org/casot/	415
	Cas‐OFFinder	Search for potential off‐target sites in a given genome or user‐defined sequence, regardless of the number of mismatches, and allow for variations in the original spacer‐adjacent motif sequence identified by Cas9.	Potential off‐target sites with complex DNA/RNA bulges may be missed. No consideration of off‐target sites due to insertions or deletions between target DNA and guide RNA sequences, and no application‐specific primers are provided.	http://www.rgenome.net/cas‐offinder/	416
	CRISPRseek	A highly flexible open source software package for identifying gRNAs that target a given input sequence and potential gRNAs while minimizing off‐target cleavage at other loci in any selected genome, generating cut scores for potential off‐target sequences.	Challenges in fully identifying and ranking potential off‐target sites.	https://bioconductor.org/packages/release/bioc/html/CRISPRseek.html	417
	CCTOP	CCTop provides an intuitive user interface with reasonable default parameters that can be easily adjusted by the user. Based on a given piece of input sequence, CCTop can identify and rank all candidate sgRNA target sites according to their off‐target quality and display the complete documentation.	Challenges in fully identifying and ranking potential off‐target sites. No ratings can be generated.	http://cctop.enzim.ttk.mta.hu	418
	CROP‐IT	For performing improved off‐target binding and cleavage site prediction, integrating genome‐wide horizontal biological information from existing Cas9 binding and cleavage datasets, outputting a scored and ranked list of potential off‐targets for improved gRNA design and more accurate prediction of Cas9 binding or cleavage sites.	Challenges in fully identifying and ranking potential off‐target sites.	http://www.adlilab.org/CROP‐IT/homepage.html	419
	CHOPCHOP; CHOPCHOP v2; CHOPCHOP v3	An intuitive and powerful tool with efficient sequence alignment algorithms for identifying CRISPR–Cas single guide RNA (sgRNA) and predicting off‐target binding of sgRNA	Challenges in fully identifying and ranking potential off‐target sites.	http://chopchop.cbu.uib.no/	420, 421, 422
	Crisflash	Ability to score potential off‐targets for all possible candidate CRISPR guide oligonucleotides.	Challenges in fully identifying and ranking potential off‐target sites.	https://github.com/crisflash	423
	CRISPOR	Predicts off‐target and can screen for efficient guide sequences using different Cas9 proteins and scoring systems.	Challenges in fully identifying and ranking potential off‐target sites.	http://crispor.org	424
	CRISTA	Predictable sgRNA cleavage efficiency for a given genomic locus.	Challenges in fully identifying and ranking potential off‐target sites.	http://crista.tau.ac.il/	425
Intracellular assay methods	WGS	The most basic whole‐genome sequencing method.	High sequencing cost and inability to detect low frequency off‐target sites.	*^‐^*	426
	HTGTS	Ability to detect off‐target loci within the genome by chromosomal translocation.	Gene editing induces fewer chromosomal translocations and is limited by chromatin accessibility.	*^‐^*	427
	BLISS	A versatile quantitative method for genome‐wide analysis of DNA double‐strand breaks, capable of detecting low‐input endogenous and exogenous DSBs.	Lack of information on the previous repair of nuclease‐induced DSBs by cellular repair mechanisms, operation time window is narrow.	*^‐^*	428
	GUIDE‐seq	One of the most commonly used off‐target assays with high sensitivity, detecting off‐target mutagenic frequencies down to 0.1%.	Limited by chromatin accessibility and cannot be used for cell types that are difficult to transfect or sensitive to cellular dsODN levels.	*^‐^*	223
	iGUIDE‐seq	An improved method for analyzing CRISPR cleavage specificity, optimized for GUIDE‐seq, provides a method for quantifying nuclease cleavage sites with the ability to distinguish between misprimers and correct primers and more accurately assess the distribution of dsDNA breaks in cells.	The sensitivity and accuracy of the assay results are limited by the efficiency and safety of the nuclease or DNA delivery.	*^‐^*	430
	DISCOVER‐seq	A universally applicable unbiased off‐target identification method that can be used with a variety of guide RNAs and CAS enzymes, providing a scientific tool for detecting in situ off‐targets in individual patient genotypes during therapeutic genome editing.	Prone to false positive reactions, low sensitivity, only DSBs at specific time intervals can be captured, limited by the difficult CHIP‐seq (chromatin immunoprecipitation) assay, and not widely used.	*^‐^*	431
	CAST‐Seq	A sensitive preclinical assay that identifies and quantitatively assesses chromosomal aberrations in hematopoietic stem cells with CRISPR–Cas nuclease targeting and off‐target activity. It is capable of identifying and quantifying unexpected chromosomal rearrangements in addition to more typical mutations at off‐target loci.	The sensitivity and accuracy of the assay results are limited by the efficiency and safety of the nuclease or DNA delivery.	*^‐^*	434
	DISCOVER‐seq	Recruitment of DNA repair factors to double‐strand breaks (DSBs) after genome editing using CRISPR nucleases for unbiased CRISPR–Cas off‐target identification in cells and tissues with low false positive rates for a variety of systems including patient‐derived cells and animal models.	DISCOVER‐seq is limited by difficult CHIP‐seq (chromatin immunoprecipitation) experiments and is not widely used.	*^‐^*	432
	DISCOVER‐Seq+	DISCOVER‐Seq+ detects up to five times more CRISPR off‐target loci than DISCOVER‐seq in immortalized cell lines, primary human cells and mice, and is the most sensitive method to date for discovering off‐target genome editing in vivo.	The sensitivity and accuracy of the assay results are limited by the efficiency and safety of the nuclease or DNA delivery.	*^‐^*	433
	GOTI	GOTI directly compares edited and nonedited cells without interference from genetic background, allows for highly sensitive detection of potential off‐target variants, and allows for a combination of experimental and computational methods to detect potential off‐target variants of any genome‐editing tool, which is critical for accurately assessing the safety of genome‐editing tools.	Expensive, time consuming and laborious.	*^‐^*	435
Extracellular assay methods	Digenome‐seq	A highly sensitive and stable unbiased method for the identification of programmable nucleases and deaminases including Cas9.	Sequencing was performed using WGS, but WGS sequencing is costly, has limited assay sensitivity, and does not enrich for nuclease cleaved sequences, requiring a large number of sequencing reads and lacking information on how cellular factors affect nuclease off‐target activity.	*^‐^*	437, 438, 439
	DIG‐seq	A genome‐wide CRISPR off‐target analysis method using chromatin DNA for in vitro nuclease cleavage with high‐throughput sequencing, with reduced off‐target effect of chromatin DNA compared with genomic DNA.	DIG‐seq is based on whole‐genome sequencing and requires a high level of coverage to achieve relatively good detection results.	*^‐^*	440
	SITE‐Seq	Enriches fragments cleared by nucleases; reduces the number of sequencing reads required.	Reads contain only one‐half of the cleavage sites; information on how cellular factors affect nuclease off‐target activity is lacking.	*^‐^*	441
	CIRCLE‐seq	A highly sensitive and unbiased sequencing strategy to identify genome‐wide off‐target CRISPR–Cas9 nucleases by selective sequencing of nuclease cleaved genomic DNA (gDNA), which can be used to identify off‐target mutations associated with cell type‐specific single nucleotide polymorphisms.	CIRCLE‐seq requires interruption of genomic DNA before concatenation into circular DNA, which is less efficient and lacks information on how cellular factors affect nuclease off‐target activity; requires large amounts of gDNA.	*^‐^*	436, 442
	CHANGE‐seq	For in vitro detection of genome‐wide activity of Cas9 to identify off‐target sites for gene editing. One‐step method with Tn5 to interrupt the genome and add connectors improves the loop formation efficiency and reduces the amount of starting genomic DNA.	The accuracy of off‐target assays is limited by cell‐specific chromatin accessibility, competition from endogenous cellular DNA–protein binding, and other factors.	*^‐^*	443
	SURRO‐seq	Sensitive and high‐throughput assessment of the off‐target effects of gene editing by pooling lentiviral off‐target libraries and deep sequencing to capture CRISPR RNA‐guided nuclease‐induced insertional deletions in cells.	The accuracy of off‐target detection is influenced by the location where the mismatch occurs and the type of mismatch.	*^‐^*	446
	Extru‐seq	A novel genome‐wide off‐target prediction method that combines the advantages of both cell‐based and in vitro approaches, showing high validation rates and retention of information about the intracellular environment, low leakage rates, and can be easily performed in clinically relevant cell types.	Difficult to detect Cas9‐mediated large deletions, chromosomal deletions and translocations, high experimental cost.	*^‐^*	447
Independent evaluation of base editor methods	EndoV‐seq	The first assay to assess the genome‐wide off‐target effect of ABE, using endonuclease V to cleave in vitro the inosine‐containing DNA strand of genomic DNA that has been deaminated by ABE, and whole‐genome sequencing of the processed DNA to identify off‐target sites.	It is limited by the fact that the same gRNA has different off‐target effects in different people due to certain differences in genome sequences of different people.	*^‐^*	449
	nDigenome‐seq	Probing the genomic specificity of the master editor (PE) can further improve PE specificity in human cells by integrating mutations in engineered Cas9 variants (eSpCas9 and Sniper Cas9) into the PE.	Dependent on in vitro Cas9 H840A incision enzyme induced SSB, only for specific types of base editing tools.	*^‐^*	125
	PEAC‐seq	A comprehensive strategy for identifying CRISPR off‐target sites and DNA translocation events in vitro and in vivo. PEAC‐seq uses prime editor to insert sequence‐optimized tags into edit sites and enrich tag regions with site‐specific primers for high‐throughput sequencing. translocations have greater genotoxicity but are typically ignored by other off‐target assays, and PEAC‐seq seq was developed to fill this gap.	The insertion efficiency of the PEAC‐seq tag may vary across pegRNAs and at different off‐targets. For each pegRNA, the sequence uniqueness of the RNA secondary structure and host genome of the insertion tag may vary.	*^‐^*	451
	TAPE‐seq	A cell‐based method that accurately and sensitively predicts genome‐wide off‐target effects of prime editor is fast, rapid and efficient when parsing large numbers of samples.	Performing TAPE‐seq in surrogate cell lines to predict off‐target sites in other cell types may result in a high off‐target prediction miss rate due to cell type‐specific activity. In addition, since pegRNA is single‐stranded, the tag sequence can form a secondary structure with adjacent RT or PBS sequences.	*^‐^*	452
	PEM‐seq	Capable of assessing the editing activity, off‐target activity, chromosomal structural abnormalities and integration of exogenous DNA fragments in Cas12f nuclease‐based gene editing systems with broad spectrum applicability, comprehensiveness and high sensitivity.	–	*^‐^*	453

Cell‐based and in vitro methods for identifying potential off‐target sites at a genome‐wide scale are used to circumvent the limitations of computational prediction tools.^411^ Cell‐based methods directly observe genome editing activity within cells. One of the primary cell‐based methods for off‐target detection is WGS (whole‐genome sequencing),^426^ HTGTS (high‐throughput whole‐genome translocation sequencing),^427^ BLISS (breaks labeling in situ and sequencing),^428^ GUIDE‐seq,^429^ iGUIDE‐seq (improved GUIDE‐seq),^430^ DISCOVER‐seq (discovery of in situ cas off‐targets and verification by sequencing),^431^, ^432^ DISCOVER‐Seq+,^433^ CAST‐Seq (chromosomal aberration analysis by single targeted linker‐mediated PCR sequencing),^434^ and GOTI (genome‐wide off‐target analysis by two‐cell embryo injection).^435^

While cell‐based approaches are vulnerable to limitations in the efficiency and safety of nuclease or DNA delivery, in vitro off‐target detection techniques directly purify the genome and perform CRISPR in vitro cutting experiments to capture the sites where off‐target cutting occurs.^436^ In vitro, off‐target detection methods include Digenome‐seq (digested genome sequencing),^437^, ^438^, ^439^ Digenome‐seq using cell‐free chromatin DNA (DIG‐seq),^440^ SITE‐seq (Selective enrichment and Identification of Tagged genomic DNA Ends by Sequencing),^441^ CIRCLE‐seq,^436^, ^442^ CHANGE‐seq (Circularization for High‐throughput Analysis of Nuclease Genome‐wide Effects by sequencing),^443^, ^444^ NucleaSeq (nuclease digestion and deep sequencing),^445^ SURRO‐seq,^446^ and Extru‐seq.^447^ Extru‐seq is a novel genome‐wide off‐target prediction method that combines the advantages of both cell‐based and in vitro approaches, showing high validation rates and retention of information about the intracellular environment, low leakage rates, which would be easily performed in clinically relevant cell types.^447^

#### 6.1.2 Off‐target assessment tool for BEs

In addition to CRISPR–Cas9‐based gene editing technology that requires off‐target detection, the application of BE‐based gene editing technology also requires the assessment and determination of genome‐wide off‐target effects. BEs can precisely edit or modulate the genome without triggering random mutations, and the off‐target effects generated mainly originate from base‐edited deaminases or lead‐edited RTs, and the off‐target sites are usually different from those of Cas9 alone, which requires an independent assessment of their genome‐wide specificity.^448^ EndoV‐seq, the first assay to assess the genome‐wide off‐target effect of ABE, used endonuclease V to cleave in vitro the inosine‐containing DNA strands of genomic DNA deaminated by ABE, and WGS of the processed DNA to identify off‐target‐sites, demonstrating that the off‐target effect of ABE is much less than that of the classical Cas9 nuclease.^449^ A Modification of Digenome‐seq for assessment of APOBEC1‐nCas9 BE (BE3) genome‐wide off‐target activity has been investigated.^448^ It has been shown that a modified version of Digenome‐seq has been optimized to detect ABE off‐target sites and to assess genome‐wide target specificity for ABE.^450^ Another study developed nickase‐based Digenome‐seq (nDigenome‐seq) to probe the genomic specificity of the PE, which can be further improved in human cells by integrating mutations from engineered Cas9 variants (eSpCas9 and Sniper Cas9) into PEs.^125^ Prime editor assisted off‐target characterization by sequencing (PEAC‐seq) is a strategy for comprehensively identifying CRISPR off‐target sites and DNA translocation events in vitro and in vivo. PEAC‐seq uses PE to insert sequence‐optimized tags into editing sites and enriches the tagged regions with site‐specific primers for high‐throughput sequencing. Translocations have greater genotoxicity but are often overlooked by other off‐target detection methods, the development of PEAC‐seq fills this gap.^451^ TAgmentation of Prime Editor sequencing (TAPE‐seq) is a cell‐based method that accurately and sensitively predicts genome‐wide off‐target effects of PE.^452^ TAPE‐seq has a lower leakage rate than other methods and identifies valid off‐target sites that are missed by other methods.^452^

Despite advances in off‐target assays, the development of credible, high‐sensitive, unbiased, and accurate ways to detect the effects of gene editing off‐target continues to pose a challenge for gene editing clinical therapies, and optimization of sgRNAs and nuclease proteins is still needed to enhance the safety of gene editing tools in clinical treatment applications.

### 6.2 Delivery

In preclinical research and clinical treatment of human diseases, CRISPR–Cas system‐based gene editing components must be delivered into cells via vectors in vitro or targeted to specific human body regions or cells for gene editing.^454^ Therefore, to apply gene editing in clinical therapy, significant challenges are to create safe and optimized methods and efficient delivery systems.^455^, ^456^ The Cas9 and sgRNA components are mainly delivered to target cells in three forms: DNA (a plasmid encoding the Cas9 protein and gRNA), RNA (Cas9 and a separate gRNA), and RNP complex.^457^, ^458^, ^459^, ^460^, ^461^ The current gene editing delivery systems mainly comprise viral and nonviral (Table 6).^14^ The viral vector system mainly includes lentivirus, adenovirus (AdV), and adeno‐associated virus (AAV) vectors.^462^ Nonviral chemical systems are usually mediated by liposomes, LNPs, and polymer carriers, such as LNPs, gold nanoparticles (AuNPs), and ionizable LNPs.^463^, ^464^, ^465^ Nonviral physical systems include electroporation and microinjection.^466^ These methods can deliver inherited material or genetic editing cargo into target cells in vitro and have unique advantages and limitations.^460^

**TABLE 6:** Main delivery methods of CRISPR–Cas system.

Delivery vehicle	Cargo	Packaging capacity	Genome integration	Immunogenicity	Pathogenicity	Advantages	Limitations	References
Viral delivery systems								
Adenoviral vectors (AdV)	DNA plasmid	∼8 kb nucleic acid	Minimal genome integration	Moderate immunogenicity	Moderate	Broad tissue orientation, ability to transduce in dividing or nondividing cells	Potentially genotoxic and carcinogenic	467
Adeno‐associated virus (AAV)	DNA plasmid	∼4.7 kb nucleic acid	Integration of low frequency vector genome sequences	Moderate immunogenicity	Low	Specific to multiple serotypes and capable of delivering ssDNA vector genome to a variety of tissues and cell types	Small packaging volume, prone to immunogenicity and hepatotoxicity	468, 469
Lentiviruses (LVs)	DNA plasmid	∼8 kb nucleic acid	Random integration of cargo genome sequences	Low immunogenicity	Low	High infection efficiency and long‐term expression of delivered genes	Possibility of insertional mutagenesis and susceptibility to gene rearrangement when delivered to gene editing systems	470
Nonviral chemical delivery systems								
Lipid‐nanoparticle (LNP)	mRNA (Cas9+sgRNA), Protein (RNP)	nM levels of Cas9 and sgRNA	Nonintegrated genome	Low immunogenicity	Low	Flexible design and wide range of histophilia (i.e., muscle, brain, liver and lung)	Low delivery efficiency	471
Gold nanoparticles (AuNP)	Protein (RNP)	nM levels of Cas9 and sgRNA	Nonintegrated genome	Low immunogenicity	Low	AuNP is considered to be relatively nontoxic and the safety of AuNP‐based in vivo delivery is high	Potential toxicity in vivo at high concentrations	472, 473
Nonviral physical delivery systems								
Microinjection	DNA plasmid, mRNA (Cas9+sgRNA), Protein (RNP)	nM levels of Cas9 and sgRNA	Integration of exogenous genes into the genome of target cells	Low immunogenicity	Low	Delivery of gene editing components to targeted locations within living cells is not limited by the various components of the cell and the ability to control the amount of delivered cargo.	Microinjection is prone to cell damage and can only target individual cells, which is inefficient for large‐scale editing	474
Electroporation	mRNA (Cas9+sgRNA), Protein (RNP)	nM levels of Cas9 and sgRNA	Nonintegrated genome	Low immunogenicity	Low	Mainly used for ex vivo gene editing, suitable for various cell types with high specificity	High voltage electroshock tends to induce cytotoxicity, cell death, and permanent membrane perforation	475

#### 6.2.1 Virus vector delivery systems

Viral vectors efficiently deliver CRISPR–Cas9 to host cells, transferring nucleic acids based on plasmids into mammalian cells for long‐term expression in vitro and in vivo. AdVs have extensive tissue tropism and do not usually integrate into the host genome, minimizing the off‐target mutagenesis risk of the CRISPR–Cas9 system.^467^ AAVs are nonimmunogenic and specific to multiple serotypes and are one of the most widely used in vivo CRISPR–Cas delivery vectors.^468^, ^476^ Small Cas9 homologs such as SaCas9 can improve the delivery efficiency of AAV vectors.^32^, ^469^, ^477^ LVs have mild immunogenicity and deliver genes that can be expressed for a long time.^470^, ^478^ The integrase‐defective LV (IDLV) overcomes the risk of insertion mutagenesis and genotoxicity problems and is a simple and feasible approach for in vivo gene therapy.^479^ Virus‐like particles are an emerging targeted delivery vehicle expressed instantaneously in vivo with little risk of genome integration.^480^

#### 6.2.2 Nonvirus delivery system

Nonviral chemical delivery systems are a promising approach to effectively avoid the potential immunogenic risks of viruses.^471^, ^481^ LNPs are a crucial tool in gene editing, pharmaceuticals, and vaccination. LNPs have been used in vivo in clinical studies for COVID‐19 mRNA vaccination and have great potential to realize gene therapy.^482^, ^483^ AuNPs and CRISPR‐Gold are delivery vectors for Cas9 RNP and donor DNA in vivo.^472^, ^484^ Microinjection and electroporation are the two main physical delivery methods. Microinjection is highly specific and reproducible.^474^ It is usually applied in vitro, where gene editing components of various molecular weights, such as RNPs, are directly delivered to the target position in living cells under a microscope.^460^ It is not restricted by various cell components and can control the number of delivery goods, effectively improving the occurrence of off‐target effects. However, it is unsuitable for large‐scale gene editing therapy.^460^ Electroporation is a widely used method for delivering proteins and nucleic acids into mammalian cells.^475^ It is mainly used for in vitro gene editing and is suitable for various cell types. It can effectively deliver CRISPR cargo such as DNAs, RNAs, or RNPs into target cells but is limited by cytotoxicity induced by high voltage shock.^458^

The challenges for successful in vivo gene editing therapies involve achieving maximal delivery to target cells, minimizing transfer to nontarget cells, reducing immunogenicity, and achieving stable expression. The development of new methods for delivering CRISPR–Cas9 systems safely and efficiently remains an active research field in gene editing, and further optimization of in vivo delivery methods for gene editing systems is necessary to improve the targeting and safety of delivery systems.

## 7 DISCUSSION

Over the past few years, as science and technology have advanced, the CRISPR–Cas system has evolved from a tool that could only delete or knock out large fragments of genes with high off‐target effects into a gene editing system that can efficiently target knockouts, mutations, and deliver more accurate gene editing of specific bases.^87^, ^124^ The development and optimization of CRISPR–Cas9, CRISPR–Cas12, CRISPR–Cas13, and other gene editing tools have greatly expanded the application range of gene editing.^75^, ^132^ Various efficient Cas9 proteins developed based on CRISPR–Cas9 and gene editing tools like BEs and plasmid editors have significantly improved gene editing efficiency and reduced off‐target events.^96^, ^128^ Developing engineered nucleases such as high‐fidelity Cas9 may overcome the potential off‐target effects of gene editing, and the smaller size of Cas proteins is beneficial for the packaging and delivery of plasmid delivery systems.^22^, ^32^ Base and PE can induce various base changes and are more secure and targeted than traditional gene editing systems.^216^ With the continuous optimization and advancement of gene editing systems, gene editing technology has shown unlimited possibilities in the preclinical research of human diseases, bringing unprecedented opportunities for disease treatment. Gene‐editing systems based on CRISPR–Cas9 have provided an alternative treatment modality to drugs for many human diseases, especially gene‐related genetic disorders that cannot be addressed by drugs and clinical procedures, which has the potential to be a safe therapeutic strategy.^8^ Gene editing technology is limited in clinical applications by off‐target editing, inefficient delivery, and difficulty in realizing complex editing. The protein structure prediction function of the protein structure prediction tool AlphaFold2 helps to develop and optimize gene editing tools. A team has utilized the protein structure prediction function of AlphaFold2 to assist protein clustering and subsequent evaluation of deaminase activity to develop a series of base editing tools, expanding the gene editing toolkit and improving the editing effect of BEs.^485^ Another study screened Cas2i3 for interaction sites with nucleic acids based on the structure prediction of AlphaFold2, and used protein engineering to combine Cas nucleic acid recognition region candidate sites to obtain an optimized version of Cas‐SF01 with high editing activity, which has similar or superior editing performance compared with SpCas9 and other Cas12 nuclease enzymes, providing efficient new tools for disease therapy and gene editing in plants and animals.^486^ With the development of artificial intelligence such as AlphaFold2, AI could realize a wide range of applications in biology and medicine.^487^ Assisted by AI, gene editing technology has the potential to overcome limiting conditions such as off‐target editing, and is expected to be more rapidly developed and applied in the clinical treatment of human diseases.

In preclinical studies, experimental animal models are essential for characterizing human diseases. Animal models of human diseases recapitulate the external pathological phenotypes and internal genetic changes that lead to morbidity to explore their pathogenesis and therapeutic effects of gene editing strategies.^140^ Small animal models represented by mice are low‐cost and easy to keep and reproduce but are limited by conditions such as small size, short lifespan, and difficulty in fully mimicking the pathological phenotypes of human diseases.^167^ Large animal models represented by NHPs are highly conserved from humans in terms of immune system and genetic changes and have a longer lifespan, which could be used to track the efficacy and safety of gene editing in vivo for several years after the treatment is administered, but the application of large animals is limited by the high cost and the difficulty of keeping and reproducing them.^167^ Currently, animal models for preclinical studies are dominated by mouse models, but because mouse models cannot fully reproduce the clinical phenotypes and genetic changes of human diseases, the development of large animal models using gene editing techniques that are more consistent with the characteristics of human diseases is expected to provide a more reductive clinical presentation and better assessment of the efficacy of gene therapy for the study of human diseases.^166^ Clarifying the in vivo stability and safety of gene editing will facilitate the clinical translation of gene editing for human diseases.

Gene editing‐based therapeutic strategies for human diseases present challenges in clinical translation such as off‐target editing and delivery vector‐induced autoimmunity. Gene editing therapies have been approved by the US FDA for clinical trials in human diseases such as hematological disorders, solid tumors, immune disorders, ophthalmic disorders, and metabolic disorders. In preclinical trials for SCD, clinical trials based on in vivo HSPC gene editing, such as Exa‐Cel, OTQ923, and EDIT‐301, have the potential to overcome the limitations of in vitro gene editing and to provide minimally invasive and cost‐effective gene therapies that show promise for use in the treatment of SCD patients in low‐ and middle‐income countries.^488^ After Exa‐Cel was renamed Casgevy, it became the first gene‐editing drug to receive US FDA approval for marketing for practical clinical application in December 2023, and the marketing and clinical application of Casgevy provides confidence in the development and marketing of future gene therapy drugs.

With the development of gene editing technology, the ethical issues of gene therapy in human trials and clinical applications have triggered multifaceted discussions.^489^ Although gene editing technology has shown unlimited potential in the treatment of disease, there are still significant technical challenges and safety concerns associated with the direct use of gene therapy in the human body.^490^ The off‐target editing of gene editing technology itself, the immune response generated by entering the human body, and the uncertainty of editing functional genes constitute the high risk of clinical applications.^213^ As gene editing technology is not available to the general public due to technological, economic, geographic, racial, and public health affordability implications, it has had an impact on equal access to gene therapy and the maintenance of social stability.^385^ Moreover, gene editing has unpredictable heritable effects on individuals and offspring, carries the risk of altering the human gene pool, and may lead to artificial and uncontrollable genetic contamination.^490^, ^491^ The World Health Organization has established an “Advisory Committee on Developing Global Standards for Governance and Oversight of Human Genome Editing.”^492^ It raises ethical issues in several areas concerning the application of gene editing to human genome editing through the Regulatory Framework for Human Gene Editing and the Human Gene Editing Recommendations, which aim to constrain the safe and ethical use of human gene editing in all countries.^493^, ^494^ Many countries have enacted laws and regulations governing the research and application of gene editing.^495^ Chinese law stipulates that medical and scientific research activities engaging in experiments on human genes and embryos shall not be contrary to ethics and morality.^496^, ^497^ Laws in various countries have established strict prohibitions against the misuse, abuse, and uncontrollable risks of gene editing in humans, to safeguard the healthy development of gene editing technologies.^498^ As discussed above, gene editing for the treatment of human diseases presents both opportunities and challenges.

## 8 CONCLUSION

Despite several remaining technical challenges, current research suggests that CRISPR–Cas9‐mediated genome editing based on CRISPR–Cas9 is a promising therapeutic approach for human diseases. Gene editing technology has shown great promise in the treatment of hematological disorders, solid tumors, immune disorders, ophthalmic diseases, and metabolic disorders through gene editing strategies. However, the application of gene editing technology in the clinical treatment of human diseases is limited by factors such as off‐target effects and the safety of delivery vectors. It is necessary to further improve gene editing technology to increase its editing efficiency, targeting, and safety, so that gene editing technology can be better applied to the clinical treatment of human diseases.

## AUTHOR CONTRIBUTIONS

*Conceptualization*: Qiang Feng, Tianjia Liu, and Dongxu Wang. *Investigation*: Qiang Feng and Dongxu Wang. *Project administration*: Tianjia Liu and Dongxu Wang. *Resources*: Dongxu Wang. *Supervision*: Tianjia Liu and Dongxu Wang. *Visualization*: Qiang Feng. *Writing—original draft*: Qiang Feng. *Writing—review and editing*: Qiang Feng, Qirong Li, Hengzong Zhou, Zhan Wang, Chao Lin, Ziping Jiang, Tianjia Liu, and Dongxu Wang. All authors have read and approved the final manuscript.

## CONFLICT OF INTEREST STATEMENT

The authors declare that they have no conflict of interest.

## ETHICS STATEMENT

Not applicable.

## Supporting information

Supporting Information

## Data Availability

The data that support the findings of this study are available in the article.
