# Supplementary material for: CRISPR technology in human diseases
Source: MedComm (2020). 2024 Jul 29;5(8):e672. doi: 10.1002/mco2.672 (PMC11286548; doi:10.1002/mco2.672)
Supplement: Supplementary file 1 — Supporting Information [file MCO2-5-e672-s001.pdf]

CRISPR Technology in Human Diseases

Qiang Feng [1,2#], Qirong Li [1#], Hengzong Zhou [1], Zhan Wang [1], Chao Lin [3], Ziping Jiang [4], Tianjia Liu [2*], Dongxu Wang [1*]

[1] *Laboratory Animal Center, College of Animal Science, Jilin University, Changchun, China*

[2] *Research and Development Centre, Baicheng Medical College, Baicheng, China*

[3] *School of grain science and technology, Jilin Business and Technology College, Changchun, China*

[4] *Department of Hand and Foot Surgery, The First Hospital of Jilin University, Changchun, China*

*** Correspondence**

Dongxu Wang, Laboratory Animal Center, College of Animal Science, Jilin University, Changchun, China.

E-mail: wang_dong_xu@jlu.edu.cn

Tianjia Liu, Research and Development Centre, Baicheng Medical College, Baicheng, China.

E-mail: ltj@bcmc.edu.cn

[#] Qiang Feng and Qirong Li contributed equally to this work.

Supplementary
Figure 1: HSPCs from patients with SCD were edited using gene editing technology and transplanted into animal models for experimental studies.
Image
created in BioRender.com.

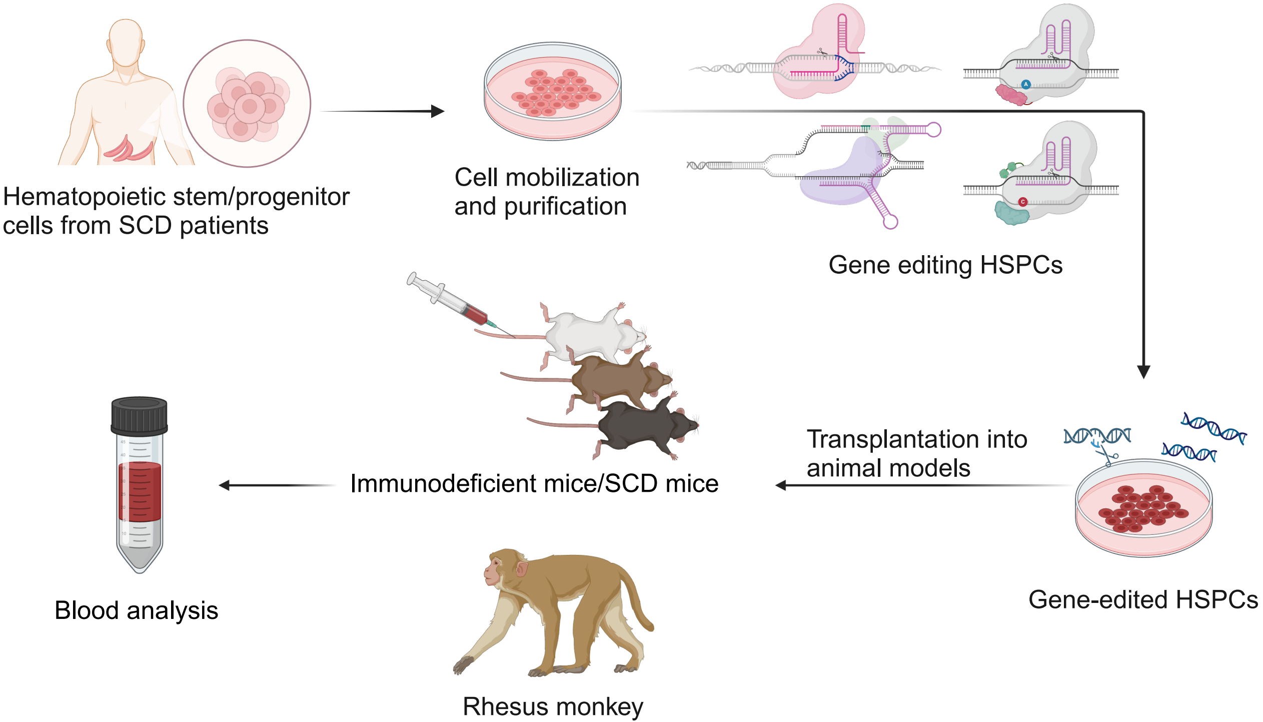

Hematopoietic stem/progenitor cells from SCD patients
Cell mobilization and purification
Gene editing HSPCs
Transplantation into animal models
Gene-edited HSPCs
Immunodeficient mice/SCD mice
Rhesus monkey
Blood analysis
